# Basal Forebrain Cholinergic Neurons: Linking Down Syndrome and Alzheimer’s Disease

**DOI:** 10.3389/fnagi.2021.703876

**Published:** 2021-07-12

**Authors:** Jose L. Martinez, Matthew D. Zammit, Nicole R. West, Bradley T. Christian, Anita Bhattacharyya

**Affiliations:** ^1^Cellular and Molecular Biology Graduate Program, University of Wisconsin, Madison, WI, United States; ^2^Waisman Center, University of Wisconsin, Madison, WI, United States; ^3^Department of Medical Physics, School of Medicine and Public Health, University of Wisconsin, Madison, WI, United States; ^4^Department of Psychiatry, School of Medicine and Public Health, University of Wisconsin, Madison, WI, United States; ^5^Department of Cellular and Regenerative Biology, School of Medicine and Public Health, University of Wisconsin, Madison, WI, United States

**Keywords:** basal forebrain cholinergic neurons, down syndrome, Alzheimer’s disease, pluripotent stem cell, neurodegeneration

## Abstract

Down syndrome (DS, trisomy 21) is characterized by intellectual impairment at birth and Alzheimer’s disease (AD) pathology in middle age. As individuals with DS age, their cognitive functions decline as they develop AD pathology. The susceptibility to degeneration of a subset of neurons, known as basal forebrain cholinergic neurons (BFCNs), in DS and AD is a critical link between cognitive impairment and neurodegeneration in both disorders. BFCNs are the primary source of cholinergic innervation to the cerebral cortex and hippocampus, as well as the amygdala. They play a critical role in the processing of information related to cognitive function and are directly engaged in regulating circuits of attention and memory throughout the lifespan. Given the importance of BFCNs in attention and memory, it is not surprising that these neurons contribute to dysfunctional neuronal circuitry in DS and are vulnerable in adults with DS and AD, where their degeneration leads to memory loss and disturbance in language. BFCNs are thus a relevant cell target for therapeutics for both DS and AD but, despite some success, efforts in this area have waned. There are gaps in our knowledge of BFCN vulnerability that preclude our ability to effectively design interventions. Here, we review the role of BFCN function and degeneration in AD and DS and identify under-studied aspects of BFCN biology. The current gaps in BFCN relevant imaging studies, therapeutics, and human models limit our insight into the mechanistic vulnerability of BFCNs in individuals with DS and AD.

## Introduction

Down syndrome (DS, trisomy 21, T21) is a complex developmental disorder that arises from trisomy of human chromosome 21 (Hsa21) (Lejeune et al., 1959) and is both a neurodevelopmental and a neurodegenerative disorder. Intellectual disability in individuals with DS ranges from mild to moderate with deficits in specific domains, including attention and memory. DS features arise as a result of uncharacteristic dosage of coding and non-coding sequences found on Hsa21. Despite its known cause and high incidence (Shin et al., 2009; Presson et al., 2013; de Graaf et al., 2015), little is known about the underlying developmental defects and degenerative outcomes that cause the characteristics of DS.

Down syndrome is also characterized by Alzheimer’s disease (AD) pathology that emerges in middle age (Scott et al., 1983; Coyle et al., 1986; Visser et al., 1997; Burt et al., 1998; Menendez, 2005; Head et al., 2012; Snyder et al., 2020). The prevalence of dementia in individuals affected by DS increases with each consecutive decade: 9% between the ages of 45 and 59 years, 18% between 50 and 54 years, and 32% between 55 and 59 years with a cumulative risk of 90% by age 65 (Zigman et al., 1996; Holland et al., 2000; Head et al., 2016; Sinai et al., 2018). In addition, the prevalence of symptomatic AD in individuals with DS reaches 90–100% by age 70, while only 11.3% of the general population have AD by the age of 65 (Fortea et al., 2020; Alzheimer’s Association, 2021). Additionally, there are sex differences, with DS males developing AD-like pathology at an earlier age than females (Zigman et al., 1996; Holland et al., 2000; Head et al., 2012). Thus, although the onset of dementia and AD in DS is beginning to be defined, it is not known what triggers the pathology nor what the earliest events in AD in DS are.

Basal forebrain cholinergic neurons (BFCNs) are a vulnerable population of neurons in both DS and AD (Yates et al., 1980; Beyreuther and Masters, 1995; Salehi et al., 2004; Baker-Nigh et al., 2015; Chen et al., 2018). BFCNs provide the primary source of cholinergic innervation to the cerebral cortex, hippocampus, and amygdala, and play a critical role in the processing of information related to cognitive function, as they are directly engaged in regulating circuits of attention and memory (Mesulam et al., 1983a; Woolf, 1991; Ballinger et al., 2016). BFCNs degenerate during aging and cell loss correlates with memory loss in old age and in individuals affected by AD (Mori, 1997; White and Ruske, 2002; Mandas et al., 2014). In DS, fewer BFCNs suggest faulty development or increased degeneration as a hallmark of reduced cognition (Casanova et al., 1985). The critical role of BFCNs in cognition, as well as their susceptibility in both DS and AD, provide a clear link to the cognitive decline in both DS and AD.

## BFCNs Are Important in AD and DS

### BFCNs Are a Unique Population of Neurons

Basal forebrain cholinergic neurons are a cluster of large neurons in the basal forebrain first described by Meynert (1872) and termed the “magnocellular basal forebrain system” (Hedreen et al., 1984) or the nucleus basalis of Meynert (NbM) in primates (Koelliker, 1896). Unlike other neuronal types, whose nuclei of origin are easy to identify, BFCNs often form dense clusters with no easily identifiable borders to justify the identification of a nucleus. Cholinergic neurons have extremely long and complex processes with a single human neuron having an estimated arborization length of >100 m (Wu et al., 2014). BFCNs express several neurotransmitter receptors that include adrenergic, glutamatergic, GABAergic, estrogen receptors, and endocannabinoids (Miettinen et al., 2002; Harkany et al., 2003; Mufson et al., 2003; Zaborszky et al., 2004; De Souza Silva et al., 2006). Their neuronal projections extend to the cerebral cortex, hippocampus, and amygdala and are the primary source of innervations to the cortex. Unlike primary sensory cortical neurons, cholinergic neurons remodel their axonal arborizations and synapses continually through the lifespan (Sarter et al., 2003; Hasselmo, 2006; Botly and De Rosa, 2009; Heys et al., 2010; Mitsushima et al., 2013; Schmitz and Duncan, 2018).

Basal forebrain cholinergic neurons are classified based on their projection targets defined in rats and non-human primates (Mesulam et al., 1983a, b; Butcher and Semba, 1989; Coppola and Disney, 2018; Solari and Hangya, 2018). Ch1 and Ch2, neurons from the medial septum and the vertical limb of the diagonal band, are the primary source of cholinergic innervation to the hippocampus. Neurons from the horizontal limb of the diagonal band, Ch3, connect to the olfactory bulb, piriform, and entorhinal cortices. These regions act as a network hub for memory, as they are the interface between the hippocampus and neocortex. Neurons in the substantia innominate/nucleus basalis, Ch4, project to the basolateral amygdala and innervate the entire neocortex. Ch1, Ch2, and Ch3 also project to orexin/hypocretin neurons in the lateral hypothalamus region of the brain (Sakurai et al., 2005). The orexinergic nucleus neurons project throughout the nervous system to mediate cognition and various physical processes (Chieffi et al., 2017). The ratio of cholinergic to non-cholinergic neuronal projections in each of these target areas varies and may affect functional connectivity. On average, the ratio is lower in the frontal area (0.3) and higher in the posterior area (0.6) (Zaborszky et al., 2015). Through this complexity, BFCNs regulate attention, memory, learning, and processing of information related to cognitive function, and so deficits in BFCN number or function can negatively impact an individual’s spatial reasoning, language, and cognition.

### BFCNs in Aging and AD

Basal forebrain cholinergic neuron dysfunction or degeneration is implicated as a driving factor for disease in a diverse range of human neurocognitive conditions and neuropsychiatric disorders including Parkinson’s disease (PD), schizophrenia, drug abuse, and AD (Détári, 2000; Conner et al., 2003; Blanco-Centurion et al., 2007; Weinberger, 2007; Jones, 2008; Kaur et al., 2008; Lin and Nicolelis, 2008; Parikh and Sarter, 2008; Goard and Dan, 2009). Strong correlation between the thinning of the Ch4 BFCNs and mild cognitive impairment of PD patients (Rong et al., 2021) suggests that loss of BFCNs contributes to the cognitive decline in PD. BFCN expression of histamine H1 receptor (H1R) is decreased in patients with schizophrenia that show negative symptoms and hallmarks of schizophrenia, such as the formation of sensorimotor gating deficit, social impairment, and anhedonia-like behavior (Cheng et al., 2021). Deleting the H1R gene in BFCNs in mice is sufficient to elicit these negative symptoms (Cheng et al., 2021), implicating a central role for this gene and these neurons in schizophrenia. Ch1, Ch2, and Ch3 BFCN projections to the orexin/hypocretin nucleus are likely linked to addiction and changes in behavior. Lesioning of BFCNs in mouse models of drug addiction suggest that interactions between BFCN- driven individual cognitive-motivational biases and the form of the drug cue encountered are involved in relapse (Pitchers et al., 2017). Together these studies, though limited, support the critical role for BFCN function in circuit function and behavior.

Basal forebrain cholinergic neurons undergo a significant level of atrophy during normal aging in mammals, including humans. This age-related degeneration is positively correlated to memory loss in old age and more prominent in individuals affected by AD (Mori, 1997; White and Ruske, 2002; Mandas et al., 2014). Cholinergic circuits are susceptible to non-pathologic age-related oxidative and inflammatory stress, which stimulates the immune system (Gamage et al., 2020). AD is defined by rapidly accelerated loss of these projection neurons (Casanova et al., 1985), with up to 90% of NbM neurons lost in familial cases of AD (Whitehouse et al., 1981). Cholinergic dysfunction correlates strongly with the progression of cognitive decline (Isacson et al., 2002; Nardone et al., 2006). Specifically, BFCN-related cognitive decline involves basocortical projection systems, septohippocampal projection systems and a loss of the high-affinity neurotrophic receptor (TrkA) expression specifically in BFCNs (Naumann et al., 2002; Mufson et al., 1996, 2003, 2004; Ginsberg et al., 2006). The loss of BFCNs during normal aging and in the pathology of AD highlight the importance of these cells in maintaining cognitive function.

Deficits in BFCNs contribute to dysfunctional neuronal circuitry in individuals with DS who have phenotypically unique behavioral patterns in language, attention and memory. Post-mortem analysis indicates that there are 29% fewer NbM neurons in adult DS compared to controls (Casanova et al., 1985). Fewer BFCNs in older DS patient samples as compared to unaffected controls suggests that loss of BFCNs contributes to memory loss, decreased spatial recognition and disturbance in language that are common areas of decline in both DS and AD (Davies and Maloney, 1976; Whitehouse et al., 1982; Coyle et al., 1983, 1986; Casanova et al., 1985; Price et al., 1986; Mufson et al., 1989, 1995; Perry et al., 1992; Bierer et al., 1995; Ballinger et al., 2016). The decreased number of these BFCNs in DS may be due to fewer cells established during brain development or due to degeneration. Understanding the vulnerability of these neurons will help us understand the underlying mechanisms of neurodegeneration in both AD and DS.

## What Is the Mechanism of BFCN Degeneration?

**FIGURE 1:**
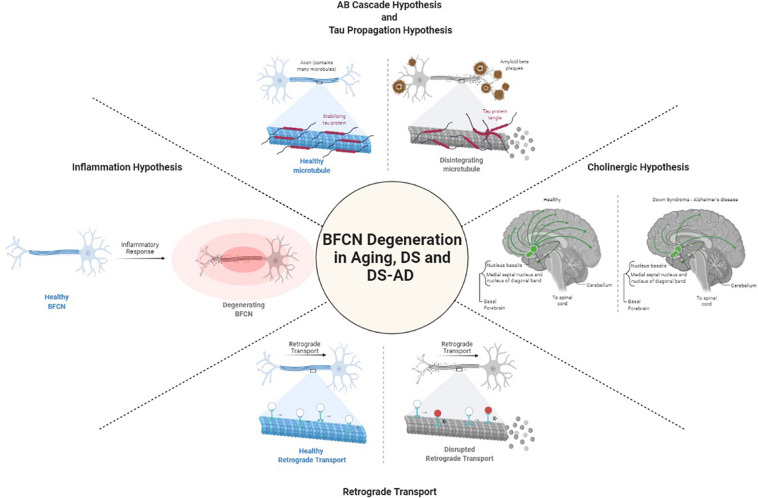
Postulated mechanisms of BFCN degeneration. Created with BioRender.

Degeneration of cholinergic neurons in the basal forebrain is strongly correlated with cognitive function. It is not known what causes the degeneration of BFCNs. Several hypotheses have been raised to define the mechanisms underlying BFCN degeneration in DS and AD including those focused on acetylcholine, amyloid-β, tau, inflammation, and retrograde transport (Figure 1). Yet, gaps in our understanding of their role specifically in BFCNs remain.

### Cholinergic Hypothesis

Because the cholinergic system is important in various forms of dementia, including AD (Davies and Maloney, 1976; Whitehouse et al., 1982; Price et al., 1986; Mufson et al., 1989; Perry et al., 1992; Bierer et al., 1995; Mufson et al., 1995; Ballinger et al., 2016), the use of choline acetyl transferase inhibitors to reverse cholinergic hypofunction in AD has been shown to facilitate memory function, albeit to a moderate degree (Ferreira-Vieira et al., 2016). The cholinergic neuronal loss in the basal forebrain is observed not only in AD, but also in PD, DS, Huntington’s disease, and other neurocognitive diseases (Aquilonius et al., 1975; Yates et al., 1980; Arendt et al., 1983; Dubois et al., 1983; Barron et al., 1987; Ferrante et al., 1987; Kato, 1989). Studies have demonstrated that cholinergic synapses are affected by amyloid-β oligomers, and this neurotoxicity is the major contributor to cognitive impairment in AD and DS (Terry et al., 1991; Selkoe, 2002; Selkoe and Hardy, 2016). These data and others led to the “cholinergic hypothesis of AD” (Coyle et al., 1983; Contestabile, 2011; Dumas and Newhouse, 2011). As discussed later, this hypothesis has fallen out of favor, but recent data should serve to revive studies on this critical system.

### Amyloid-β Cascade Hypothesis

The amyloid-β cascade hypothesis was advanced by the finding of a pathogenic mutation in the APP gene (encoded on Hsa21), which indicated that APP metabolism and amyloid-β deposition were the primary events in AD (Hardy and Allsop, 1991; Selkoe, 1991). APP is cleaved by two different proteolytic processes: the amyloidogenic (β pathway, pathogenic) that results in production of insoluble amyloid-β and the non-amyloidogenic (α pathway, non-pathogenic) pathway (Liu et al., 2019) that does not produce insoluble amyloid-β. It is well established that high concentrations of amyloid-β protein are neurotoxic to neurons, causing atrophy of the axons and dendrites leading to neuronal death (Yankner et al., 1990). Normally the small amount of amyloid-β that is produced *via* the β pathway is cleared by the immune system, but APP mutations such as Lys670Asn/Met671Leu (Swedish) can direct more amyloidogenic proteolysis (Yan and Vassar, 2014; Zhou et al., 2018). Similarly, individuals with a rare familial trait known as duplication of APP (Dup-APP), also develop early onset AD (Rovelet-Lecrux et al., 2006, 2007; Sleegers et al., 2006; Kasuga et al., 2009; Thonberg et al., 2011; Hooli et al., 2012; McNaughton et al., 2012; Swaminathan et al., 2012; Wiseman et al., 2015). Thus, APP and amyloid-β have a causative role in AD.

The additional copy of Hsa21-encoded APP in DS may be a driving factor for the emergence of AD in individuals with DS by increasing amyloid-β. Likewise, individuals with a partial trisomy of chromosome 21 that lack an additional copy of APP do not develop AD (Prasher et al., 1998; Korbel et al., 2009). While these data suggest a key role of APP in the development of AD in DS, recent studies from DS models show the important role of APP in the amyloidogenic aspects of AD but challenge the notion that increased APP levels are solely responsible for DS-associated AD pathogenesis (Wiseman et al., 2015, 2018; Ovchinnikov et al., 2018). Additional genes on Hsa21 may regulate the course of AD in DS individuals, but further work is required to elucidate their role and importance.

### Tau Propagation Hypothesis

The tau propagation hypothesis focuses on the appearance of neurofibrillary tangles (NFTs) and misfolded tau that propagates through the brain in a prion-like way, eventually spreading throughout the brains of AD patients (Frost et al., 2009). Tau proteins stabilize the microtubules that act as a highway for the transportation of cargo in dendrites and axons (Clavaguera et al., 2009; Frost et al., 2009). Tau is encoded by approximately 352 residues and alternative splicing of exons 2, 3, and 10 results in six isoforms. The balance between isoforms regulates cellular processes (Goedert et al., 1989; Andreadis et al., 1992). An equilibrium between two isoforms (3R and 4R) may be important in preventing the formation of tau aggregates, a common feature in AD pathology. Hyperphosphorylated tau proteins form helical filaments, which aggregate to form NFTs (Grundke-Iqbal et al., 1986; Kosik et al., 1986; Nukina and Ihara, 1986), a pathological feature of AD (Braak and Braak, 1996; Braak et al., 1999).

Individuals with DS show the formation of NFTs as early as 30 years of age (Lott and Head, 2019; Perez et al., 2019; Gomez et al., 2020). Tau phosphorylation and the appearance of NFTs in DS may be regulated by two Hsa21 genes, APP and DYRK1A. DYRK1A phosphorylates APP at the Thr668 residue, which leads to an increase in activated APP. The resulting p-APP phosphorylates the Thr212 residue of tau, resulting in pTau that is implicated in AD pathology (Alonso et al., 2010, 2018). A biomarker study revealed that individuals with DS had decreased amyloid-β over time, while the plasma level of tau and NFT increased leading to a reduction in basal forebrain volume (Mengel et al., 2020; Schmitz et al., 2020). Thus, in addition to APP, tau phosphorylation may be dysregulated in DS and specifically in BFCNs.

### Inflammation Hypothesis

Inflammation occurs in the brains of individuals with AD and DS patients as a response to neuritic plaques and NFTs (Wilcock, 2012). The inflammatory response is predominantly mediated by microglial cells, brain-specific macrophages in the central nervous system (CNS) that make up about 15% of all brain cells (Clayton et al., 2017). Microglial inflammatory responses have been identified as potentially playing an important role in the development of AD pathology (Clayton et al., 2017; Kinney et al., 2018; Chen and Mobley, 2019). In AD patients a two to five-fold increase in the concentration of aggregated microglia near neurons with NFTs may indicate higher activity of microglial cells in AD (Calsolaro and Edison, 2016). Amyloid-β has a synergistic effect with the cytokine activation of microglia (Meda et al., 1995). It is through the CD36-TLR4-TLR6 receptor complex and the NLRP3 inflammatory complex that amyloid-β can bind to microglia cells, release inflammation factors and elicit immune responses (Heneka et al., 2013; Sheedy et al., 2013). Levels of inflammatory factors such as TNF-a, IL-1β, TGF-β, IL-12, and IL-8 correlate with AD and increased levels in the CNS have also been implicated in increased damage in brains of AD patients (Michaud et al., 2013). Biomarkers in DS plasma show consistently higher levels of amyloid-β and IL-1β (Startin et al., 2019). Similarly, in DS, IL-1β and TGF-β induce Hsa21 proteases ADAMTS1 and ADAMTS5 in the CNS. The ADAMTS proteins are of interest because they are involved in neurodegeneration (Gurses et al., 2016). Inflammation likely plays a major role in AD in DS, but the mechanism is currently under investigation. Additionally, it is not clear whether BFCNs are affected by inflammation.

### Retrograde Transport

Basal forebrain cholinergic neurons depend on nerve growth factor (NGF) and brain derived neurotrophic factors (BDNF) for their survival and function (Fahnestock and Shekari, 2019). Both neurotrophic factors are retrogradely transported from BFCN targets. The precursor for NGF (proNGF; pNGF) binds to the NGF receptors TrkA and p75NTR, binding with higher affinity to p75NTR while mature NGF binds more strongly to the TrkA receptor (Fahnestock and Shekari, 2019). BFCNs express both receptors, which are activated by pNGF to elicit TrkA-dependent pathways of survival and growth through MAPK and Akt-mTOR. However, inactivation or imbalance of TrkA leads to activation of p75NTR-dependent apoptotic pathways, such as JNK. TrkA is also increasingly lost in mild cognitive impairment and AD (Fahnestock and Shekari, 2019). Studies modeling aging with embryonic rat basal forebrain neurons in culture have shown the axonal transport of NGF and BDNF are impaired with age, suggesting a vulnerability of BFCNs in aging as well as in age-related disorders such as AD (Budni et al., 2015).

In DS, it has been speculated that the additional copy of APP on Hsa21 has a downstream impact on the retrograde transport of neurotrophins. Using mouse models, overexpression of APP hyperactivates Rab5, a key regulator of endosome fusion and trafficking, leading to abnormally large endosomes, which normally carry the NGF signal retrogradely (Xu et al., 2016). There are two explanations as to how hyperactivation of Rab5 impairs neuronal trophic signaling. First, the enlarged Rab5 endosomes may have a difficult time moving retrogradely within the axon, thus resulting in a net decrease in NGF delivery to the soma (Xu et al., 2018). Alternatively, the increase Rab5 activation can promote premature delivery of trophic signals to late endosomes/lysosomes, resulting in early degeneration of NGF/TrkA signaling (Zhang et al., 2013; Xu et al., 2018). Both of these possibilities would lead to decreased trophic signaling and support of BFCNs, resulting in neuronal death. No studies have assessed retrograde transport in human DS BFCNs and so we do not know whether similar mechanisms are in play.

Here, we summarize the predominant mechanisms that have been raised that may underlie specifically BFCN pathology. However, other characteristics of T21 cells may also be important in BFCN pathology. For example, oxidative stress has long been implicated in DS (Lott et al., 2006; Perluigi and Butterfield, 2012) and, in fact, treatment of aged Ts65Dn mice with vitamin E reduced oxidation levels and decreased cholinergic neuron pathology in the basal forebrain (Lockrow et al., 2009). Human studies are needed to test whether these results translate to humans.

It is also possible deficient autophagy contributes to BFCN pathology (Colacurcio et al., 2018). Assessment of human T21 cortical neurons has recently emerged (Botté et al., 2020) and so it will be important for future studies to define vesicle trafficking in BFCNs as well as cortical neurons.

## Imaging Reveals Vulnerability of BFCNs in AD and DS

Although there are multiple vulnerable populations of neurons in various brain regions, the classic model of AD pathology progression postulates that the initial accumulation of pTau, and later amyloid-β accumulation, in the entorhinal cortices leads to the degeneration process that spreads to the temporoparietal cortex over time in a stage-like fashion (Braak and Braak, 1991; Thal et al., 2002; Fernandez and Lopez, 2020). Imaging studies of AD models support this hypothesis, as they indicate that the accumulation of pTau and amyloid-β in certain brain regions reflect the local neural vulnerability that spreads overtime (Davies and Maloney, 1976; Whitehouse et al., 1981; Arendt et al., 1985; Mesulam et al., 2004; Mattson and Magnus, 2006; Geula et al., 2008; Braak and Del Tredici, 2011; Saxena and Caroni, 2011; Khan et al., 2014; Baker-Nigh et al., 2015; Grothe et al., 2018; Sepulcre et al., 2018; Hanseeuw et al., 2019). Yet, recent imaging studies have started to challenge this model. A longitudinal study using cerebrospinal fluid (CSF) and MRI data from the Alzheimer’s Disease Neuroimaging Initiative (ADNI) identified neurodegeneration of the NbM in abnormal and normal groups defined by previously validated CSF pTau/amyloid-β ratios (Fernandez-Cabello et al., 2020). Two non-overlapping and well powered data sets from the ADNI, along with whole-brain regression models show that the relationship between NbM volumes and neurodegeneration is specific to regions of the entorhinal cortex and the perirhinal cortices (Fernandez-Cabello et al., 2020). These results suggest a model in which amyloid-β pathology in the ascending BFCN projections from NbM first spreads to the entorhinal cortex and then to the temporoparietal neurodegeneration typically attributed to the earliest stages of AD. The degeneration of the BFCN projection system as an early event in AD pathology highlights the susceptibility of these neurons to early pathology and later downstream impacts on other vulnerable populations, challenging the current notion that the entorhinal cortex is upstream of this event. There is a clear need for additional studies exploring the initial events in AD pathology to better understand the early disease stage vulnerable populations.

There are limited human studies in DS that focus on BFCNs as a vulnerable neuronal population, highlighting the critical need for more neuropathological and imaging studies. However, within the last several years, large studies using positron emission tomography (PET) to characterize the preclinical progression of AD in DS have emerged using the AT(N) (amyloid/tau/neurodegeneration) disease research framework (Jack et al., 2018; Fortea et al., 2020; Rafii et al., 2020). Furthermore, no DS studies with PET focus on imaging of BFCNs during preclinical AD. The Alzheimer’s Biomarker Consortium – Down Syndrome (ABC-DS) is an ongoing longitudinal study aimed to better understand AD progression in DS by characterizing AD biomarker change in one of the world’s largest DS research cohorts (Handen et al., 2020). With PET imaging, a pattern of early and prominent amyloid-β retention was identified in the dorsal and ventral striatum (Handen et al., 2012); a pattern which has also been observed in other forms of early-onset AD (Klunk et al., 2007; Remes et al., 2008; Villemagne et al., 2009; Bateman et al., 2012). Apart from the striatum, the cortical retention of amyloid-β in DS has an identical pattern to late-onset AD, with amyloid-β increasing at longitudinal rates of 3–4% annually (Lao et al., 2017; Tudorascu et al., 2019; Zammit et al., 2020a, 2021).

Imaging of NFTs with PET is a more recent addition to the field of AD research, but its use in DS is very limited. Through the Down Syndrome Biomarker Initiative, an early DS study with a relatively small sample size demonstrated that increased NFT burden was highly associated with cognitive impairment (Rafii et al., 2017). A study from the ABC-DS with a large sample size identified that NFT retention in DS conforms to the conventional Braak staging of NFT pathology, with the earliest evidence of NFTs in the entorhinal cortex and hippocampus (Tudorascu et al., 2020). NFT PET studies in DS have also been limited to cross-sectional analyses, and longitudinal measurements are needed to characterize the annual rates of NFT progression and the latency period between the onset of amyloid-β and NFTs. PET imaging of glucose metabolic change is also envisioned as a proxy measurement for neurodegeneration in DS.

In DS, glucose hypometabolism has been observed with local increases in amyloid-β throughout regions implicated in AD (Lao et al., 2018). Glucose hypometabolism in the frontal cortex, anterior cingulate, posterior cingulate, parietal cortex, precuneus, and temporal cortex were also highly associated with worsening cognitive performance evaluated using measures of episodic memory (Zammit et al., 2020b), which have been validated as sensitive indicators of the transition between preclinical and prodromal AD in DS (Hartley et al., 2020). In addition, PET measurement of glucose metabolism was capable of distinguishing cases of MCI-DS and AD from cognitively stable DS, suggesting it as a sensitive marker of neurodegeneration (Zammit et al., 2020b). Increased imaging of AD has shown the progression of biomarkers between DS and late-onset AD are very similar, but future studies would require close examination of BFCNs *in vivo* to identify the link between AD biomarkers and BFCN degeneration.

## Interventions for AD and DS

Current FDA approved pharmacological interventions for AD are limited. There are five approved AD medications; donepezil, galantamine, rivastigmine, memantine, and a combination of donepezil and memantine (Alzheimer’s Association, 2019). Donepezil, galantamine, and rivastigmine are acetylcholinesterase inhibitors, while memantine is a non-competitive low-affinity NMDA receptor open-channel blocker that also affects glutamatergic transmission (Yiannopoulou and Papageorgiou, 2020). Recent work has focused on designing experimental drugs targeting specific points of the pathophysiological mechanism of AD that include amyloid-β, pTau metabolism, mitochondrial dysfunction, oxidative stress, and inflammation. Most, if not all, have proven clinically unsuccessful, with Donepezil being the last FDA approved AD drug in 2010. It is important to note that most of the AD drugs are acetylcholinesterase inhibitors that reduce the breakdown of acetylcholine released from BFCNs. Much like donepezil, galantamine works by inhibiting acetylcholinesterase in a reversible and selective manner while rivastigmine is a pseudo-irreversible inhibitor of both acetylcholinesterase and butyrylcholinesterase. These inhibitors can mitigate the memory deficits associated with aging and AD (Rusted, 1994; van Reekum et al., 1997; Du et al., 2018). However, their effects appear to be transient, as they only show efficacy during the first year of administration, with further memory decline occurring later. In the AD2000 study, a large “real life” trial on the impact of regular use of donepezil, AD patients treated with donepezil did not show significant benefits compared to placebo in progression of disability at 3 years of treatment, rendering this approach a symptomatic relief with marginal benefits (Bentham et al., 2004; Du et al., 2018). These results led to the cholinergic hypothesis and targeting of the cholinergic pathway falling out of favor in the AD research community (Cacabelos, 2007). Yet, it is clear that the cholinergic system is of high importance in AD and DS and that BFCNs remain a relevant cell population and potential therapeutic target.

Despite the prevalence of AD in DS patients, individuals with DS have been traditionally excluded from most clinical trials of anti-dementia drugs (Strydom et al., 2018). Cholinergic therapies have been advocated for DS to ameliorate dysfunctional neuronal circuitry (Kishnani et al., 2001). The available AD-related pharmacologic therapies offer minimal usefulness in symptom reduction and fail to stop or slow down disease progression (Areosa and Sherriff, 2003; Cacabelos, 2007; Folch et al., 2018; Tayebati et al., 2019). Yet, combined treatment with cholinesterase inhibitors and memantine have also been used to ameliorate both cognitive and behavioral issues in AD and DS. A longitudinal study of 310 people with DS and AD indicated that those undergoing cholinesterase inhibitor treatment had comparable outcomes, improved cognition and behavior, to those with sporadic AD (Eady et al., 2018). More interestingly, individuals with DS treated with either a single cholinesterase or in combination had a median survival rate of ∼5.6 years after diagnosis, an improvement compared to those who did not take medication who had a median survival rate of ∼3.4 years (Eady et al., 2018). Not only did these results show that modulating the cholinergic system can improve cognition, but it can also have a significant impact on the length of survival for DS individuals diagnosed with AD. Thus, the cholinergic system and BFCNs in particular warrant further investigation as a potential therapeutic target in DS.

In addition to the FDA approved medications for AD, additional experimental therapies are being considered to ameliorate cognitive decline or AD onset in DS. Inspired by improvement and protective mechanisms against neurodegeneration in *Caenorhabditis elegans* models of PD, treatment of DS induced pluripotent stem cell (iPSC)-derived neurons with N-butylidenephthalide reduced amyloid-β aggregates and NFTs (Chang et al., 2015). This amyloid-β scavenger is a promising therapy to target the proteopathy of AD that leads to BFCN deficits. Rapamycin rescues molecular pathways associated with abnormal mTOR phosphorylation and ameliorates the rate of neurodegeneration in DS mouse models, improving their cognition (Tramutola et al., 2018). Lastly, the use of Fluoxetine, a widely used antidepressant, in a DS mouse model at an early postnatal age showed promise in increasing neurogenesis and reducing learning deficits (Guidi et al., 2013). However, further human studies focused on BFCNs are needed, as this field heavily relies on animal models.

## Modeling AD in DS and BFCNs

### Mouse Models: Do They Recapitulate BFCN Pathology?

Mouse models of DS enable experimental approaches that are not feasible in humans, such as the study of disease progression in a regulated environment, intervention trials, validation of imaging results, and also permit gene-gene interaction studies of Hsa21-specific DS genes (Hamlett et al., 2016; Herault et al., 2017). Of the 225 protein coding genes found on Hsa21, 166 are conserved in three regions in mice, murine (mmu) chromosome 10, 16, and 17 (Hattori et al., 2000; Akeson et al., 2001). Mouse models of DS have provided evidence of the influence of individual genes on Hsa21 that lead to deficits in BFCNs (Kiss et al., 1989; Sweeney et al., 1989; Coyle et al., 1991; Cooper et al., 2001; Hunter et al., 2004; Salehi et al., 2006; Ash et al., 2014; Kelley et al., 2014a, 2019; Powers et al., 2016, 2017).

The vast majority of the aging and AD studies in DS have been conducted on the Ts65Dn mouse, the prevalent model of DS for many years (Davisson et al., 1993; Reeves et al., 1995). Developed in the early 1990s by Muriel Davisson, this model contains 120 orthologs of Hsa21 protein encoding genes *via* a segmental trisomy of mmu 16 (Davisson et al., 1993). The aneuploidy in the Ts65Dn mouse is not lethal as in the Ts16 mouse model, but their lifespan is shorter than diploid mice (Sanders et al., 2009). However, 25% of trisomic genes in Ts65Dn are not Hsa21 orthologs, and 45% of Hsa21 orthologs are not trisomic (Zhao and Bhattacharyya, 2018). Thus, the Ts65Dn model has genetic limitations as an age-related DS and AD pathology model. Nonetheless Ts65Dn mice do show several relevant deficits including progressive memory decline, hippocampal abnormalities, increased APP production, and adult-onset degeneration of BFCNs, locus coeruleus neurons, and noradrenergic cortical innervations (Hamlett et al., 2016). Sex differences have been described in the Ts65Dn model; female Ts65Dn mice show a decrease in BFCN number as well as a smaller NbM region area as compared to males by 34 and 20%, respectively (Kelley et al., 2014b). No human studies have assessed sex differences in BFCNs and so we do not know how well these results translate to humans.

Similar to the Ts65Dn model, the Tc1 mouse model shows many relevant phenotypes including abnormalities in learning, memory, and synaptic plasticity (Gardiner et al., 2003). The Tc1 mouse model is trisomic for 212 of the Hsa21 protein coding genes (Hamlett et al., 2016). In contrast to Ts65Dn mice, Tc1 mice also exhibit higher levels of S100B calcium-binding protein, AMPK, and the mTORC1 proteins RAPTOR and downstream kinase P70S6, crucial regulators of cellular metabolism and aging. The Ts1Cje model, which contains a shorter Mmu16 trisomy than the Ts65Dn mouse, and the Ts2Cje model, whose chromosomal rearrangement of the Ts65Dn genome caused a translocation to Mmu12 forming a Robertsonian chromosome, show similar phenotypes to Ts65Dn. Both Ts1Cje and Ts2Cje mice exhibit oxidative stress, tau hyper-phosphorylation, mitochondrial dysfunction, and show some learning and memory deficits, and ultimately BFCN degeneration similar to the processes identified in Ts65Dn mice (Hamlett et al., 2016).

MS1Ts65 mouse models of DS contain only a small fragment of Hsa21 orthologs in comparison to other models (Duchon et al., 2011). They contain approximately 33 orthologs of Hsa21 genes within the genetic segment ranging from APP to Sod1 (Sago et al., 1998). With complete trisomy of all Hsa21 syntenic regions, the Mmu10− Dp(10)1Yey/+ (Ts1Yey), Mmu17− Dp(16)1Yey/+ (Ts2Yey), and Mmu16− Dp(17)1Yey/+ (Ts3Yey) triple aneuploid mouse model is the most complete model of DS to date (Li et al., 2007; Yu et al., 2010). Ts3Yey mice have similar brain morphology to Ts65Dn mice and confirm the genetic basis for behavioral and morphological phenotypes, thus offering promise for developing more appropriate and complete mouse models for DS in the future (Duchon et al., 2011; Hamlett et al., 2016).

Basal forebrain cholinergic neuron neuropathology is apparent in Ts65Dn mouse models of AD in DS. Age-related degeneration starts around 6–8 months of age, with significant BFCN cell body atrophy at 6 months and major loss at age 8 and 10 months (Contestabile et al., 2006; Hamlett et al., 2016). In addition, major deficits in both choline acetyltransferase (ChAT) and NGF receptor TrkA in BFCNs are detectable at these ages (Contestabile et al., 2006). At a later age, increased neurochemical markers, including inflammatory markers and APP cleavage products, suggest continual progression of AD neuropathy in Ts65Dn mice (Contestabile et al., 2006). Although this study highlights the critical role of BFCNs in the progression of AD in DS, it is crucial to develop both animal and human models that capture the full trisomy in DS to better study this neuropathology.

Targeting the cholinergic pathway as a therapeutic strategy has been carried out in Ts65Dn mice. Maternal choline supplementation and gene expression analysis of laser capture microdissection (LCM)-captured CA1 pyramidal neurons in maternal choline supplemented Ts65Dn mice offspring at 6 months (prior to BFCN degeneration) and 11 months (post BFCN degeneration) of age had improved spatial and recognition memory task performance as compared to their littermate controls (Lockrow et al., 2011), highlight the importance of cholinergic levels in DS for healthy neural circuits (Alldred et al., 2018, 2019). Memantine treatment in these mice resulted in increased expression of the neurotrophic factor BDNF in the frontal cortex and hippocampus (Lockrow et al., 2011). Thus, mouse models of DS provide proof of principle that cholinergic therapies may be successful.

### Human Models

The incomplete genetic recapitulation of Hsa21 in mouse models, and trisomy of non-Hsa21 orthologs, likely influences the effects of orthologous Hsa21 genes and, more importantly, may cause genetic consequences and downstream cellular and behavioral characteristics that are not relevant to DS. Thus, there is need for analysis of human cells from individuals with DS to have complete trisomy of Hsa21 (Herault et al., 2017; Zhao and Bhattacharyya, 2018). In addition, the failures of clinical trials in AD for therapeutic targets based on mouse models argues for the use of human patient-derived cells in target identification and drug screening.

The discovery of reprogramming factors to generate iPSCs from adult somatic cell types opened the doors to derive PSCs from individuals with specific genetic and non-genetic disorders, including DS (Thomson et al., 1998; Takahashi et al., 2007; Yu et al., 2007; Park et al., 2008b). iPSCs can model human neural development by mimicking *in vivo* spatial and temporal cues during brain development *in vitro* (Tao and Zhang, 2016) and enable the establishment of functionally specialized neural subtypes (Park et al., 2008a; Chou et al., 2012; MacLean et al., 2012; Mou et al., 2012; Briggs et al., 2013; Jiang et al., 2013; Lu et al., 2013; Weick et al., 2013; Chen et al., 2014; Hibaoui et al., 2014; Pipino et al., 2014; Huo et al., 2018). iPSCs are thus a useful model system to study DS and AD (Li et al., 2012; Murray et al., 2015; Ovchinnikov et al., 2018; Real et al., 2018).

Isogenic control iPSCs are important research tools to distinguish the consequences of T21 from human genetic variation. The generation of isogenic euploids can result from culture-induced spontaneous loss of the extra Hsa21 (Park et al., 2008a; MacLean et al., 2012). Alternatively, 2–4% of DS cases are mosaic individuals in which their somatic cells are mosaic for T21 (Papavassiliou et al., 2009; Murray et al., 2015). By taking advantage of cellular mosaicis, isogenic T21 and euploid iPSCs can be derived from the same individual (Weick et al., 2013; Murray et al., 2015; Gough et al., 2020).

Isogenic cells can be generated by inducing chromosome loss (Real et al., 2018). In addition, various methods have been used to genetically correct the gene dose of the T21 by eliminating or selectively mutating specific genes. Alternatively, others have taken a candidate gene approach to selectively reduce the gene dose using CRISPR/Cas9-mediated gene manipulation (Park et al., 2008a). Full chromosomal correction of the gene dose imbalance has been accomplished using XIST-mediated (Jiang et al., 2013) or TKNEO-mediated silencing of the trisomic chromosome in iPSCs (Li et al., 2012) and ZSCAN-induced elimination of the extra chromosome (Amano et al., 2015). These strategies may enable elucidation of the genetic and cellular consequences of T21.

### iPSC to BFCN

Much of the research done using T21 iPSCs derived from individuals with DS has been to understand cortical development and pathology (Weick et al., 2013; Huo et al., 2018; Real et al., 2018). T21 iPSC-derived cortical neurons showed impairment in synaptic activity, as well as compensatory responses to oxidative stress (Shi et al., 2012; Briggs et al., 2013; Weick et al., 2013; Sobol et al., 2019).

**FIGURE 2:**
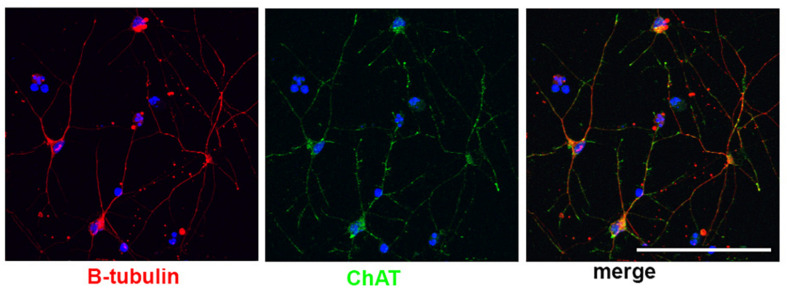
Human stem cell derived BFCNs. Immunofluorescence of BFCNs derived from human iPSCs showing neurons (β-tubulin, red), choline acetyltransferase (ChAT, green), and merged image showing co-expression. Scale bar, 100 um.

Little work has been done to model BFCNs with iPSCs in DS and AD. Basal forebrain neurons (including BFCNs and GABAergic interneurons) originate in neurogenic areas of the most ventral regions of the telencephalon, the medial ganglionic eminences (MGE) and preoptic area (POA) (Sussel et al., 1999; Brazel et al., 2003). Patterning of the MGE is dependent on a sonic hedgehog (SHH) signaling gradient for ventralization of the neural tube (Xu et al., 2005; Gulacsi and Anderson, 2006; Li et al., 2009). MGE progenitors express the transcription factor NKX2.1, whose expression is regulated by SHH (Du et al., 2008; Xu et al., 2008, 2010). Despite the known development of BFCNs from the MGE, few differentiation protocols have been established to generate BFCNs from hPSCs (Bissonnette et al., 2011; Liu et al., 2013a; Duan et al., 2014; Hu et al., 2016), and many result in mixed populations of cells. The most robust technique (Liu et al., 2013a; Hu et al., 2016) relies on an initial ventralization patterning with SHH and addition of NGF to allow for the survival, differentiation and maturation of BFCN and yields ∼90% progenitors expressing NKX2.1. Moreover, ∼40% of NKX2.1+ cells co-express OLIG2 and ∼15% of NKX2.1+ cells also express ISLET1, which are ventral markers and are both important for BFCN development (Wang and Liu, 2001; Furusho et al., 2006). Approximately 40% of the resulting neurons express ChAT, the enzyme responsible for biosynthesis of the neurotransmitter acetylcholine and a mature BFCN marker (Hu et al., 2016; Figure 2). Others have also successfully used small molecules to pattern BFCNs resulting in efficiencies ranging from 15 up to 80% (Liu et al., 2013b; Yue et al., 2015; Hu et al., 2016; Muñoz et al., 2020). Although the yields of BFCNs are good, the mixed neuronal cultures leave room for improvement in the established BFCN protocols.

The promising strategies to derive BFCNs from PSCs indicates the use of isogenic iPSCs will enable us to define markers of dysfunction, aging, and degeneration in these cells to reveal molecular signatures and signaling pathways underlying BFCN degeneration in DS and DS-AD. One of the many advantages of PSC models is the retention of the human genetic background by establishing patient specific iPSCs (Brazel et al., 2003; Li et al., 2009). However, through the reprogramming process iPSCs lose many of the aging markers of the somatic donor cells (Xu et al., 2005; Gulacsi and Anderson, 2006). The resulting iPSCs also share transcriptional and functional profile similarities to those in fetal development, making it difficult to study age-related diseases. Thus, generating hPSC-derived neurons that mirror those in the adult and aging brain is essential for neurodegenerative disease modeling using hPSCs. Further, the results have the potential to inform our understanding of the vulnerability of BFCNs in DS, AD, PD (Brazel et al., 2003), amyotrophic lateral sclerosis (Li et al., 2009), progressive supranuclear palsy (Xu et al., 2005; Gulacsi and Anderson, 2006), and olivopontocerebellar atrophy (Xu et al., 2010).

## Summary

With an increased life expectancy of DS individuals, it is important to study the cellular and molecular mechanisms that underlie neurodegeneration and AD in DS (Sawada et al., 2008; Duncan, 2011; Baker and Petersen, 2018). Here, we have raised the need to address the significant gaps in the understanding of the vulnerability of BFCNs in aging and disease by highlighting the critical role of BFCNs in cognition, the vulnerability of BFCNs in animal models of DS and AD and indications that BFCNs degeneration may be one of the earliest events in AD and DS neuropathology.

## Author Contributions

AB and JM contributed to concept and approach of the review. JM collected and organized the research articles and wrote the first draft of the manuscript. MZ and NW wrote and edited sections of the manuscript. All authors contributed to manuscript revision, read, and approved the submitted version.

## Conflict of Interest

The authors declare that the research was conducted in the absence of any commercial or financial relationships that could be construed as a potential conflict of interest.

## References

[B1] Alzheimer’s Association (2021). 2021 Alzheimer’s disease facts and figures. *Alzheimers Dement.* 17 327–406. 10.1002/alz.12328 33756057

[B2] Akeson E. C. Lambert J. P. Narayanswami S. Gardiner K. Bechtel L. J. Davisson M. T. (2001). Ts65Dn – localization of the translocation breakpoint and trisomic gene content in a mouse model for Down syndrome. *Cytogenet. Cell Genet.* 93 270–276. 10.1159/000056997 11528125

[B3] Alldred M. J. Chao H. M. Lee S. H. Beilin J. Powers B. E. Petkova E. (2018). CA1 pyramidal neuron gene expression mosaics in the Ts65Dn murine model of Down syndrome and Alzheimer’s disease following maternal choline supplementation. *Hippocampus* 28 251–268. 10.1002/hipo.22832 29394516 PMC5874173

[B4] Alldred M. J. Chao H. M. Lee S. H. Beilin J. Powers B. E. Petkova E. (2019). Long-term effects of maternal choline supplementation on CA1 pyramidal neuron gene expression in the Ts65Dn mouse model of Down syndrome and Alzheimer’s disease. *FASEB J.* 33 9871–9884. 10.1096/fj.201802669rr 31180719 PMC6704451

[B5] Alonso A. D. Cohen L. S. Corbo C. Morozova V. ElIdrissi A. Phillips G. (2018). Hyperphosphorylation of Tau associates with changes in its function beyond microtubule stability. *Front. Cell. Neurosci.* 12:338. 10.3389/fncel.2018.00338 30356756 PMC6189415

[B6] Alonso A. D. Di Clerico J. Li B. Corbo C. P. Alaniz M. E. Grundke-Iqbal I. (2010). Phosphorylation of tau at Thr212, Thr231, and Ser262 combined causes neurodegeneration. *J. Biol. Chem.* 285 30851–30860. 10.1074/jbc.m110.110957 20663882 PMC2945578

[B7] Alzheimer’s Association (2019). *FDA-Approved Treatments for Alzheimer’s.* Chicago, IL: Alzheimer’s Association.

[B8] Amano T. Jeffries E. Amano M. Ko A. C. Yu H. Ko M. S. (2015). Correction of Down syndrome and Edwards syndrome aneuploidies in human cell cultures. *DNA Res.* 22 331–342. 10.1093/dnares/dsv016 26324424 PMC4596399

[B9] Andreadis A. Brown W. M. Kosik K. S. (1992). Structure and novel exons of the human-TAU gene. *Biochemistry* 31 10626–10633. 10.1021/bi00158a027 1420178

[B10] Aquilonius S. M. Eckernås S. A. Sundwall A. (1975). Regional distribution of choline acetyltransferase in the human brain: changes in Huntington’s chorea. *J. Neurol. Neurosurg. Psychiatry.* 38 669–677. 10.1136/jnnp.38.7.669 125784 PMC1083246

[B11] Arendt T. Bigl V. Arendt A. Tennstedt A. (1983). Loss of neurons in the nucleus basalis of Meynert in Alzheimer’s disease, paralysis agitans and Korsakoff’s Disease. *Acta Neuropathol.* 61 101–108. 10.1007/bf00697388 6637393

[B12] Arendt T. Bigl V. Tennstedt A. Arendt A. (1985). Neuronal loss in different parts of the nucleus basalis is related to neuritic plaque formation in cortical target areas in Alzheimer’s disease. *Neuroscience* 14 1–14. 10.1016/0306-4522(85)90160-5 3974875

[B13] Areosa S. A. Sherriff F. (2003). Memantine for dementia. *Cochrane Database Systemat. Rev.* 3:CD003154. 10.1002/14651858.CD003154 12535459

[B14] Ash J. A. Velazquez R. Kelley C. M. Powers B. E. Ginsberg S. D. Mufson E. J. (2014). Maternal choline supplementation improves spatial mapping and increases basal forebrain cholinergic neuron number and size in aged Ts65Dn mice. *Neurobiol. Dis.* 70 32–42. 10.1016/j.nbd.2014.06.001 24932939 PMC4133151

[B15] Baker D. J. Petersen R. C. (2018). Cellular senescence in brain aging and neurodegenerative diseases: evidence and perspectives. *J. Clin. Investig.* 128 1208–1216. 10.1172/jci95145 29457783 PMC5873891

[B16] Baker-Nigh A. Vahedi S. Davis E. G. Weintraub S. Bigio E. H. Klein W. L. (2015). Neuronal amyloid-beta accumulation within cholinergic basal forebrain in ageing and Alzheimer’s disease. *Brain* 138 1722–1737. 10.1093/brain/awv024 25732182 PMC4542619

[B17] Ballinger E. C. Ananth M. Talmage D. A. Role L. W. (2016). Basal forebrain cholinergic circuits and signaling in cognition and cognitive decline. *Neuron* 91 1199–1218. 10.1016/j.neuron.2016.09.006 27657448 PMC5036520

[B18] Barron S. A. Mazliah J. Bental E. (1987). Sympathetic cholinergic dysfunction in amyotrophic lateral sclerosis. *Acta Neurol. Scand.* 75 62–63. 10.1111/j.1600-0404.1987.tb07890.x 3577669

[B19] Bateman R. J. Xiong C. Benzinger T. L. Fagan A. M. Goate A. Fox N. C. (2012). Clinical and biomarker changes in dominantly inherited Alzheimer’s disease. *N. Engl. J. Med.* 367 795–804. 22784036 10.1056/NEJMoa1202753 PMC3474597

[B20] Bentham P. Gray R. Raftery J. Hills R. Sellwood E. Courtney C. (2004). Long-term donepezil treatment in 565 patients with Alzheimer’s disease (AD2000): randomised double-blind trial. *Lancet* 363 2105–2115. 10.1016/s0140-6736(04)16499-4 15220031

[B21] Beyreuther K. Masters C. L. (1995). Neurodegeneration and dementia – Alzheimers-disease as a model. *Arzneimittel Forschung* 45 347–350. 7763324

[B22] Bierer L. M. Haroutunian V. Gabriel S. Knott P. J. Carlin L. S. Purohit D. P. (1995). Neurochemical correlates of dementia severity in Alzheimer’s disease: relative importance of the cholinergic deficits. *J. Neurochem.* 64 749–760. 10.1046/j.1471-4159.1995.64020749.x 7830069

[B23] Bissonnette C. J. Lyass L. Bhattacharyya B. J. Belmadani A. Miller R. J. Kessler J. A. (2011). The controlled generation of functional basal forebrain cholinergic neurons from human embryonic stem cells. *Stem Cells* 29 802–811. 10.1002/stem.626 21381151 PMC3107131

[B24] Blanco-Centurion C. Gerashchenko D. Shiromani P. J. (2007). Effects of saporin-induced lesions of three arousal populations on daily levels of sleep and wake. *J. Neurosci.* 27 14041–14048. 10.1523/jneurosci.3217-07.2007 18094243 PMC2975593

[B25] Botly L. C. De Rosa E. (2009). Cholinergic deafferentation of the neocortex using 192 IgG-saporin impairs feature binding in rats. *J. Neurosci.* 29 4120–4130. 10.1523/jneurosci.0654-09.2009 19339607 PMC6665388

[B26] Botté A. Lainé J. Xicota L. Heiligenstein X. Fontaine G. Kasri A. (2020). Ultrastructural and dynamic studies of the endosomal compartment in Down syndrome. *Acta Neuropathol. Commun.* 8:89. 10.1186/s40478-020-00956-z PMC7315513 32580751

[B27] Braak E. Griffing K. Arai K. Bohl J. Bratzke H. Braak H. (1999). Neuropathology of Alzheimer’s disease: what is new since A. Alzheimer? *Eur. Arch. Psychiatry Clin. Neurosci.* 249(Suppl. 3) 14–22. 10.1007/pl00014168 10654095

[B28] Braak H. Braak E. (1991). Neuropathological stageing of Alzheimer-related changes. *Acta Neuropathol.* 82 239–259. 10.1007/bf00308809 1759558

[B29] Braak H. Braak E. (1996). Evolution of the neuropathology of Alzheimer’s disease. *Acta Neurol. Scand. Suppl.* 165 3–12. 8740983 10.1111/j.1600-0404.1996.tb05866.x

[B30] Braak H. Del Tredici K. (2011). The pathological process underlying Alzheimer’s disease in individuals under thirty. *Acta Neuropathol.* 121 171–181. 10.1007/s00401-010-0789-4 21170538

[B31] Brazel C. Y. Romanko M. J. Rothstein R. P. Levison S. W. (2003). Roles of the mammalian subventricular zone in brain development. *Prog. Neurobiol.* 69 49–69. 10.1016/s0301-0082(03)00002-9 12637172

[B32] Briggs J. A. Sun J. Shepherd J. Ovchinnikov D. A. Chung T. L. Nayler S. P. (2013). Integration-free induced pluripotent stem cells model genetic and neural developmental features of down syndrome etiology. *Stem Cells* 31 467–478. 10.1002/stem.1297 23225669

[B33] Budni J. Bellettini-Santos T. Mina F. Garcez M. L. Zugno A. I. (2015). The involvement of BDNF, NGF and GDNF in aging and Alzheimer’s disease. *Aging Dis.* 6 331–341. 10.14336/ad.2015.0825 26425388 PMC4567216

[B34] Burt D. B. Loveland K. A. Primeaux-Hart S. Chen Y. W. Phillips N. B. Cleveland L. A. (1998). Dementia in adults with Down syndrome: diagnostic challenges. *Am. J. Ment. Retard.* 103 130–145. 9779281 10.1352/0895-8017(1998)103<0130:DIAWDS>2.0.CO;2

[B35] Butcher L. L. Semba K. (1989). Reassessing the cholinergic basal forebrain – Nomenclature schemata and concepts. *Trends Neurosci.* 12 483–485. 10.1016/0166-2236(89)90102-1 2480660

[B36] Cacabelos R. (2007). Donepezil in Alzheimer’s disease: from conventional trials to pharmacogenetics. *Neuropsychiatr. Dis. Treat.* 3 303–333. 19300564 PMC2654795

[B37] Calsolaro V. Edison P. (2016). Neuroinflammation in Alzheimer’s disease: current evidence and future directions. *Alzheimers Dement.* 12 719–732. 10.1016/j.jalz.2016.02.010 27179961

[B38] Casanova M. F. Walker L. C. Whitehouse P. J. Price D. L. (1985). Abnormalities of the nucleus basalis in Down’s syndrome. *Ann. Neurol.* 18 310–313. 10.1002/ana.410180306 2932050

[B39] Chang C. Y. Chen S. M. Lu H. E. Lai S. M. Lai P. S. Shen P. W. (2015). N-butylidenephthalide attenuates Alzheimer’s disease-like cytopathy in Down syndrome induced pluripotent stem cell-derived neurons. *Sci. Rep.* 5:8744. 10.1038/srep08744 PMC4348654 25735452

[B40] Chen C. Jiang P. Xue H. Peterson S. E. Tran H. T. McCann A. E. (2014). Role of astroglia in Down’s syndrome revealed by patient-derived human-induced pluripotent stem cells. *Nat. Commun.* 5:4430. 10.1038/ncomms5430 PMC4109022 25034944

[B41] Chen X.-Q. Mobley W. C. (2019). Exploring the pathogenesis of Alzheimer disease in basal forebrain cholinergic neurons: converging insights from alternative hypotheses. *Front. Neurosci.* 13:446. 10.3389/fnins.2019.0044 PMC6514132 31133787

[B42] Chen X. Q. Sawa M. Mobley W. C. (2018). Dysregulation of neurotrophin signaling in the pathogenesis of Alzheimer disease and of Alzheimer disease in Down syndrome. *Free Rad. Biol. Med.* 114 52–61. 10.1016/j.freeradbiomed.2017.10.341 29031834 PMC5748266

[B43] Cheng L. Xu C. Wang L. An D. Jiang L. Zheng Y. (2021). Histamine H(1) receptor deletion in cholinergic neurons induces sensorimotor gating ability deficit and social impairments in mice. *Nat. Commun.* 12:1142. 10.1038/s41467-021-21476-x PMC7893046 33602941

[B44] Chieffi S. Carotenuto M. Monda V. Valenzano A. Villano I. Precenzano F. (2017). Orexin system: the key for a healthy life. *Front. Physiol.* 8:357. 10.3389/fphys.2017.00357 28620314 PMC5450021

[B45] Chou S. T. Byrska-Bishop M. Tober J. M. Yao Y. VanDorn D. Opalinska J. B. (2012). Trisomy 21-associated defects in human primitive hematopoiesis revealed through induced pluripotent stem cells. *Proc. Natl. Acad. Sci. U.S.A.* 109 17573–17578. 10.1073/pnas.1211175109 23045704 PMC3491490

[B46] Clavaguera F. Bolmont T. Crowther R. A. Abramowski D. Frank S. Probst A. (2009). Transmission and spreading of tauopathy in transgenic mouse brain. *Nat. Cell Biol.* 11 909–U325. 19503072 10.1038/ncb1901 PMC2726961

[B47] Clayton K. A. Van Enoo A. A. Ikezu T. (2017). Alzheimer’s disease: the role of microglia in brain homeostasis and proteopathy. *Front. Neurosci.* 11:680. 10.3389/fnins.2017.00680 29311768 PMC5733046

[B48] Colacurcio D. J. Pensalfini A. Jiang Y. Nixon R. A. (2018). Dysfunction of autophagy and endosomal-lysosomal pathways: roles in pathogenesis of Down syndrome and Alzheimer’s Disease. *Free Rad. Biol. Med.* 114 40–51. 10.1016/j.freeradbiomed.2017.10.001 28988799 PMC5748263

[B49] Conner J. M. Culberson A. Packowski C. Chiba A. A. Tuszynski M. H. (2003). Lesions of the Basal forebrain cholinergic system impair task acquisition and abolish cortical plasticity associated with motor skill learning. *Neuron* 38 819–829. 10.1016/s0896-6273(03)00288-5 12797965

[B50] Contestabile A. (2011). The history of the cholinergic hypothesis. *Behav. Brain Res.* 221 334–340. 10.1016/j.bbr.2009.12.044 20060018

[B51] Contestabile A. Fila T. Bartesaghi R. Contestabile A. Ciani E. (2006). Choline acetyltransferase activity at different ages in brain of Ts65Dn mice, an animal model for Down’s syndrome and related neurodegenerative diseases. *J. Neurochem.* 97 515–526. 10.1111/j.1471-4159.2006.03769.x 16539660

[B52] Cooper J. D. Salehi A. Delcroix J. D. Howe C. L. Belichenko P. V. Chua-Couzens J. (2001). Failed retrograde transport of NGF in a mouse model of Down’s syndrome: reversal of cholinergic neurodegenerative phenotypes following NGF infusion. *Proc. Natl. Acad. Sci. U.S.A.* 98 10439–10444. 10.1073/pnas.181219298 11504920 PMC56979

[B53] Coppola J. J. Disney A. A. (2018). Is there a canonical cortical circuit for the cholinergic system? Anatomical differences across common model systems. *Front. Neural Circuits* 12:8. 10.3389/fncir.2018.00008 29440996 PMC5797555

[B54] Coyle J. T. Oster-Granite M. L. Gearhart J. D. (1986). The neurobiologic consequences of Down syndrome. *Brain Res. Bull.* 16 773–787. 10.1016/0361-9230(86)90074-2 2875770

[B55] Coyle J. T. Oster-Granite M. L. Reeves R. Hohmann C. Corsi P. Gearhart J. (1991). Down syndrome and the trisomy 16 mouse: impact of gene imbalance on brain development and aging. *Res. Publ. Assoc. Res. Nerv. Ment. Dis.* 69 85–99. 1825889

[B56] Coyle J. T. Price D. L. DeLong M. R. (1983). Alzheimer’s disease: a disorder of cortical cholinergic innervation. *Science* 219 1184–1190. 10.1126/science.6338589 6338589

[B57] Davies P. Maloney A. J. (1976). Selective loss of central cholinergic neurons in Alzheimer’s disease. *Lancet* 2:1403. 10.1016/s0140-6736(76)91936-x 63862

[B58] Davisson M. T. Schmidt C. Reeves R. H. Irving N. G. Akeson E. C. Harris B. S. (1993). Segmental trisomy as a mouse model for Down syndrome. *Prog. Clin. Biol. Res.* 384 117–133. 8115398

[B59] de Graaf G. Buckley F. Skotko B. G. (2015). Estimates of the live births, natural losses, and elective terminations with Down syndrome in the United States. *Am. J. Med. Genet. Part A* 167 756–767. 10.1002/ajmg.a.37001 25822844

[B60] De Souza Silva M. A. Dolga A. Pieri I. Marchetti L. Eisel U. L. Huston J. P. (2006). Cholinergic cells in the nucleus basalis of mice express the N-methyl-D-aspartate-receptor subunit NR2C and its replacement by the NR2B subunit enhances frontal and amygdaloid acetylcholine levels. *Genes Brain Behav.* 5 552–560. 10.1111/j.1601-183x.2006.00206.x 17010101

[B61] Détári L. (2000). Tonic and phasic influence of basal forebrain unit activity on the cortical EEG. *Behav. Brain Res.* 115 159–170. 10.1016/s0166-4328(00)00256-4 11000418

[B62] Du T. Xu Q. Ocbina P. J. Anderson S. A. (2008). NKX2.1 specifies cortical interneuron fate by activating Lhx6. *Development* 135 1559–1567. 10.1242/dev.015123 18339674

[B63] Du X. G. Wang X. Y. Geng M. Y. (2018). Alzheimer’s disease hypothesis and related therapies. *Transl. Neurodegener.* 7:7. 10.1186/s40035-018-0107-y PMC5789526 29423193

[B64] Duan L. Bhattacharyya B. J. Belmadani A. Pan L. Miller R. J. Kessler J. A. (2014). Stem cell derived basal forebrain cholinergic neurons from Alzheimer’s disease patients are more susceptible to cell death. *Mol. Neurodegener.* 9:3. 10.1186/1750-1326-9-3 24401693 PMC3896712

[B65] Dubois B. Ruberg M. Javoy-Agid F. Ploska A. Agid Y. (1983). A subcortico-cortical cholinergic system is affected in Parkinson’s disease. *Brain Res.* 288 213–218. 10.1016/0006-8993(83)90096-3 6661617

[B66] Duchon A. Raveau M. Chevalier C. Nalesso V. Sharp A. J. Herault Y. (2011). Identification of the translocation breakpoints in the Ts65Dn and Ts1Cje mouse lines: relevance for modeling down syndrome. *Mammal. Genome* 22 674–684. 10.1007/s00335-011-9356-0 21953411 PMC3224224

[B67] Dumas J. A. Newhouse P. A. (2011). The cholinergic hypothesis of cognitive aging revisited again: cholinergic functional compensation. *Pharmacol. Biochem. Behav.* 99 254–261. 10.1016/j.pbb.2011.02.022 21382398 PMC3114182

[B68] Duncan G. W. (2011). The aging brain and neurodegenerative diseases. *Clin. Geriatr. Med.* 27 629–644. 22062445 10.1016/j.cger.2011.07.008

[B69] Eady N. Sheehan R. Rantell K. Sinai A. Bernal J. Bohnen I. (2018). Impact of cholinesterase inhibitors or memantine on survival in adults with Down syndrome and dementia: clinical cohort study. *Br. J. Psychiatry* 212 155–160. 10.1192/bjp.2017.21 29486820

[B70] Fahnestock M. Shekari A. (2019). ProNGF and neurodegeneration in Alzheimer’s disease. *Front. Neurosci.* 13:129. 10.3389/fnins.2019.00129 30853882 PMC6395390

[B71] Fernandez M. M. Lopez V. M. (2020). Simultaneous auditory-visual support in grammatical intervention in subjects with intellectual disability. *Rev. Educ.* 389 115–140.

[B72] Fernandez-Cabello S. Kronbichler M. Van Dijk K. R. A. Goodman J. A. Spreng R. N. Schmitz T. W. (2020). Basal forebrain volume reliably predicts the cortical spread of Alzheimer’s degeneraion. *Brain* 143:1009. 10.1093/brain/awaa012 PMC7092749 32203580

[B73] Ferrante R. J. Beal M. F. Kowall N. W. Richardson E. P. Jr. Martin J. B. (1987). Sparing of acetylcholinesterase-containing striatal neurons in Huntington’s disease. *Brain Res.* 411 162–166. 10.1016/0006-8993(87)90694-9 2955849

[B74] Ferreira-Vieira T. H. Guimaraes I. M. Silva F. R. Ribeiro F. M. (2016). Alzheimer’s disease: targeting the cholinergic system. *Curr. Neuropharmacol.* 14 101–115. 26813123 10.2174/1570159X13666150716165726 PMC4787279

[B75] Folch J. Busquets O. Ettcheto M. Sánchez-López E. Castro-Torres R. D. Verdaguer E. (2018). Memantine for the treatment of dementia: a review on its current and future applications. *J. Alzheimers Dis.* 62 1223–1240. 10.3233/jad-170672 29254093 PMC5870028

[B76] Fortea J. Vilaplana E. Carmona-Iragui M. Benejam B. Videla L. Barroeta I. (2020). Clinical and biomarker changes of Alzheimer’s disease in adults with Down syndrome: a cross-sectional study. *Lancet* 395 1988–1997. 32593336 10.1016/S0140-6736(20)30689-9 PMC7322523

[B77] Frost B. Jacks R. L. Diamond M. I. (2009). Propagation of tau misfolding from the outside to the inside of a cell. *J. Biol. Chem.* 284 12845–12852. 10.1074/jbc.m808759200 19282288 PMC2676015

[B78] Furusho M. Ono K. Takebayashi H. Masahira N. Kagawa T. Ikeda K. (2006). Involvement of the Olig2 transcription factor in cholinergic neuron development of the basal forebrain. *Dev. Biol.* 293 348–357. 10.1016/j.ydbio.2006.01.031 16537079

[B79] Gamage R. Wagnon I. Rossetti I. Childs R. Niedermayer G. Chesworth R. (2020). Cholinergic modulation of glial function during aging and chronic neuroinflammation. *Front. Cell. Neurosci.* 14:577912. 10.3389/fncel.2020.577912 33192323 PMC7594524

[B80] Gardiner K. Fortna A. Bechtel L. Davisson M. T. (2003). Mouse models of Down syndrome: how useful can they be? Comparison of the gene content of human chromosome 21 with orthologous mouse genomic regions. *Gene* 318 137–147. 10.1016/s0378-1119(03)00769-8 14585506

[B81] Geula C. Nagykery N. Nicholas A. Wu C.-K. (2008). Cholinergic neuronal and axonal abnormalities are present early in aging and in Alzheimer disease. *J. Neuropathol. Exp. Neurol.* 67 309–318. 10.1097/nen.0b013e31816a1df3 18379437 PMC3243760

[B82] Ginsberg S. D. Che S. Wuu J. Counts S. E. Mufson E. J. (2006). Down regulation of trk but not p75NTR gene expression in single cholinergic basal forebrain neurons mark the progression of Alzheimer”s disease. *J. Neurochem.* 97 475–487. 10.1111/j.1471-4159.2006.03764.x 16539663

[B83] Goard M. Dan Y. (2009). Basal forebrain activation enhances cortical coding of natural scenes. *Nat. Neurosci.* 12 1444–1449. 10.1038/nn.2402 19801988 PMC3576925

[B84] Goedert M. Spillantini M. G. Jakes R. Rutherford D. Crowther R. A. (1989). Multiple isoforms of human microtubule-associated protein-tau - sequences and localization in neurofibrillary tangles of Alzheimers-disease. *Neuron* 3 519–526. 10.1016/0896-6273(89)90210-9 2484340

[B85] Gomez W. Morales R. Maracaja-Coutinho V. Parra V. Nassif M. (2020). Down syndrome and Alzheimer’s disease: common molecular traits beyond the amyloid precursor protein. *Aging* 12 1011–1033. 10.18632/aging.102677 31918411 PMC6977673

[B86] Gough G. O’Brien N. L. Alic I. Goh P. A. Yeap Y. J. Groet J. (2020). Modeling Down syndrome in cells: from stem cells to organoids. *Prog. Brain Res.* 251 55–90. 10.1016/bs.pbr.2019.10.003 32057312

[B87] Grothe M. J. Sepulcre J. Gonzalez-Escamilla G. Jelistratova I. Schöll M. Hansson O. (2018). Molecular properties underlying regional vulnerability to Alzheimer’s disease pathology. *Brain* 141 2755–2771. 30016411 10.1093/brain/awy189 PMC6113636

[B88] Grundke-Iqbal I. Iqbal K. Tung Y. C. Quinlan M. Wisniewski H. M. Binder L. I. (1986). Abnormal phosphorylation of the microtubule-associated protein tau (tau) in Alzheimer cytoskeletal pathology. *Proc. Natl. Acad. Sci. U.S.A.* 83 4913–4917. 10.1073/pnas.83.13.4913 3088567 PMC323854

[B89] Guidi S. Stagni F. Bianchi P. Ciani E. Ragazzi E. Trazzi S. (2013). Early pharmacotherapy with fluoxetine rescues dendritic pathology in the Ts65Dn mouse model of down syndrome. *Brain Pathol.* 23 129–143. 10.1111/j.1750-3639.2012.00624.x 22817700 PMC8028975

[B90] Gulacsi A. Anderson S. A. (2006). Shh maintains Nkx2.1 in the MGE by a Gli3-independent mechanism. *Cereb. Cortex* 16(Suppl. 1) i89–i95. 16766713 10.1093/cercor/bhk018

[B91] Gurses M. S. Ural M. N. Gulec M. A. Akyol O. Akyol S. (2016). Pathophysiological function of ADAMTS enzymes on molecular mechanism of Alzheimer’s disease. *Aging Dis.* 7:479. 10.14336/ad.2016.0111 27493839 PMC4963191

[B92] Hamlett E. D. Boger H. A. Ledreux A. Kelley C. M. Mufson E. J. Falangola M. F. (2016). Cognitive impairment, neuroimaging, and Alzheimer neuropathology in mouse models of down syndrome. *Curr. Alzheimer Res.* 13 35–52. 10.2174/1567205012666150921095505 26391050 PMC5034871

[B93] Handen B. L. Cohen A. D. Channamalappa U. Bulova P. Cannon S. A. Cohen W. I. (2012). Imaging brain amyloid in nondemented young adults with Down syndrome using Pittsburgh compound B. *Alzheimers Dement.* 8 496–501. 10.1016/j.jalz.2011.09.229 23102120 PMC3532743

[B94] Handen B. L. Lott I. T. Christian B. T. Schupf N. OBryant S. Mapstone M. (2020). The Alzheimer’s biomarker consortium-Down syndrome: rationale and methodology. *Alzheimers Dement.* 12:e12065. 10.1002/dad2.12065 PMC7396809 32775597

[B95] Hanseeuw B. J. Betensky R. A. Jacobs H. I. Schultz A. P. Sepulcre J. Becker J. A. (2019). Association of amyloid and tau with cognition in preclinical Alzheimer disease: a longitudinal study. *JAMA Neurol.* 76 915–924. 31157827 10.1001/jamaneurol.2019.1424 PMC6547132

[B96] Hardy J. Allsop D. (1991). Amyloid deposition as the central event in the aetiology of Alzheimer’s disease. *Trends Pharmacol. Sci.* 12 383–388. 10.1016/0165-6147(91)90609-v 1763432

[B97] Harkany T. Härtig W. Berghuis P. Dobszay M. B. Zilberter Y. Edwards R. H. (2003). Complementary distribution of type 1 cannabinoid receptors and vesicular glutamate transporter 3 in basal forebrain suggests input-specific retrograde signalling by cholinergic neurons. *Eur. J. Neurosci.* 18 1979–1992. 10.1046/j.1460-9568.2003.02898.x 14622230

[B98] Hartley S. L. Handen B. L. Devenny D. Tudorascu D. Piro-Gambetti B. Zammit M. D. (2020). Cognitive indicators of transition to preclinical and prodromal stages of Alzheimer’s disease in Down syndrome. *Alzheimers Dement.* 12:e12096. 10.1002/dad2.12096 PMC7507534 32995465

[B99] Hasselmo M. E. (2006). The role of acetylcholine in learning and memory. *Curr. Opinion Neurobiol.* 16 710–715. 10.1016/j.conb.2006.09.002 17011181 PMC2659740

[B100] Hattori M. Fujiyama A. Taylor T. D. Watanabe H. Yada T. Park H. S. (2000). The DNA sequence of human chromosome 21. *Nature* 405 311–319. 10830953 10.1038/35012518

[B101] Head E. Lott I. T. Wilcock D. M. Lemere C. A. (2016). Aging in Down syndrome and the development of Alzheimer’s disease neuropathology. *Curr. Alzheimer Res.* 13 18–29. 10.2174/1567205012666151020114607 26651341 PMC4948181

[B102] Head E. Powell D. Gold B. T. Schmitt F. A. (2012). Alzheimer’s disease in Down syndrome. *Eur. J. Neurodegener. Dis.* 1 353–364. 25285303 PMC4184282

[B103] Hedreen J. C. Struble R. G. Whitehouse P. J. Price D. L. (1984). Topography of the magnocellular basal forebrain system in human brain. *J. Neuropathol. Exp. Neurol.* 43 1–21. 10.1097/00005072-198401000-00001 6319616

[B104] Heneka M. T. Kummer M. P. Stutz A. Delekate A. Schwartz S. Vieira-Saecker A. (2013). NLRP3 is activated in Alzheimer’s disease and contributes to pathology in APP/PS1 mice. *Nature* 493 674–678. 10.1038/nature11729 23254930 PMC3812809

[B105] Herault Y. Delabar J. M. Fisher E. M. C. Tybulewicz V. L. J. Yu E. Brault V. (2017). Rodent models in Down syndrome research: impact and future opportunities. *Dis. Models Mech.* 10 1165–1186. 10.1242/dmm.029728 28993310 PMC5665454

[B106] Heys J. G. Giocomo L. M. Hasselmo M. E. (2010). Cholinergic modulation of the resonance properties of stellate cells in layer II of medial entorhinal cortex. *J. Neurophysiol.* 104 258–270. 10.1152/jn.00492.2009 20445030 PMC2904208

[B107] Hibaoui Y. Grad I. Letourneau A. Sailani M. R. Dahoun S. Santoni F. A. (2014). Modelling and rescuing neurodevelopmental defect of Down syndrome using induced pluripotent stem cells from monozygotic twins discordant for trisomy 21. *EMBO Mol. Med.* 6 259–277. 10.1002/emmm.201302848 24375627 PMC3927959

[B108] Holland A. J. Hon J. Huppert F. A. Stevens F. (2000). Incidence and course of dementia in people with Down’s syndrome: findings from a population-based study. *J. Intellect. Disabil. Res.* 44(Pt 2) 138–146. 10.1046/j.1365-2788.2000.00263.x 10898377

[B109] Hooli B. Mohapatra G. Mattheisen M. Parrado A. Roehr J. Shen Y. (2012). Role of common and rare APP DNA sequence variants in Alzheimer disease. *Neurology* 78 1250–1257. 10.1212/wnl.0b013e3182515972 22491860 PMC3324321

[B110] Hu Y. Qu Z. Y. Cao S. Y. Li Q. Ma L. Krencik R. (2016). Directed differentiation of basal forebrain cholinergic neurons from human pluripotent stem cells. *J. Neurosci. Methods* 266 42–49. 10.1016/j.jneumeth.2016.03.017 27036311

[B111] Hunter C. L. Bachman D. Granholm A. C. (2004). Minocycline prevents cholinergic loss in a mouse model of Down’s syndrome. *Ann. Neurol.* 56 675–688. 10.1002/ana.20250 15468085

[B112] Huo H. Q. Qu Z. Y. Yuan F. Ma L. X. Yao L. Xu M. (2018). Modeling Down syndrome with patient iPSCs reveals cellular and migration deficits of GABAergic neurons. *Stem Cell Rep.* 10 1251–1266. 10.1016/j.stemcr.2018.02.001 29526735 PMC5998838

[B113] Isacson O. Seo H. Lin L. Albeck D. Granholm A. C. (2002). Alzheimer’s disease and Down’s syndrome: roles of APP, trophic factors and ACh. *Trends Neurosci.* 25 79–84. 10.1016/s0166-2236(02)02037-4 11814559

[B114] Jack C. R. Jr. Bennett D. A. Blennow K. Carrillo M. C. Dunn B. Haeberlein S. B. (2018). NIA-AA research framework: toward a biological definition of Alzheimer’s disease. *Alzheimers Dement.* 14 535–562. 10.1016/j.jalz.2018.02.018 29653606 PMC5958625

[B115] Jiang J. Jing Y. C. Cost G. J. Chiang J. C. Kolpa H. J. Cotton A. M. (2013). Translating dosage compensation to trisomy 21. *Nature* 500 296–300. 23863942 10.1038/nature12394 PMC3848249

[B116] Jones B. E. (2008). Modulation of cortical activation and behavioral arousal by cholinergic and orexinergic systems. *Ann. N. Y. Acad. Sci.* 1129 26–34. 10.1196/annals.1417.026 18591466

[B117] Kasuga K. Shimohata T. Nishimura A. Shiga A. Mizuguchi T. Tokunaga J. (2009). Identification of independent APP locus duplication in Japanese patients with early-onset Alzheimer disease. *J. Neurol. Neurosurg. Psychiatry.* 80 1050–1052. 10.1136/jnnp.2008.161703 19684239

[B118] Kato T. (1989). Choline acetyltransferase activities in single spinal motor neurons from patients with amyotrophic lateral sclerosis. *J. Neurochem.* 52 636–640. 10.1111/j.1471-4159.1989.tb09167.x 2911033

[B119] Kaur S. Junek A. Black M. A. Semba K. (2008). Effects of ibotenate and 192IgG-saporin lesions of the nucleus basalis magnocellularis/substantia innominata on spontaneous sleep and wake states and on recovery sleep after sleep deprivation in rats. *J. Neurosci.* 28 491–504. 10.1523/jneurosci.1585-07.2008 18184792 PMC6670515

[B120] Kelley C. M. Ginsberg S. D. Alldred M. J. Strupp B. J. Mufson E. J. (2019). Maternal choline supplementation alters basal forebrain cholinergic neuron gene expression in the Ts65Dn mouse model of down syndrome. *Dev. Neurobiol.* 79 664–683. 10.1002/dneu.22700 31120189 PMC6756931

[B121] Kelley C. M. Powers B. E. Velazquez R. Ash J. A. Ginsberg S. D. Strupp B. J. (2014a). Maternal choline supplementation differentially alters the basal forebrain cholinergic system of young-adult Ts65Dn and disomic mice. *J. Comp. Neurol.* 522 1390–1410. 10.1002/cne.23492 24178831 PMC3959592

[B122] Kelley C. M. Powers B. E. Velazquez R. Ash J. A. Ginsberg S. D. Strupp B. J. (2014b). Sex differences in the cholinergic basal forebrain in the Ts65Dn mouse model of Down syndrome and Alzheimer’s disease. *Brain Pathol.* 24 33–44. 10.1111/bpa.12073 23802663 PMC4220609

[B123] Khan U. A. Liu L. Provenzano F. A. Berman D. E. Profaci C. P. Sloan R. (2014). Molecular drivers and cortical spread of lateral entorhinal cortex dysfunction in preclinical Alzheimer’s disease. *Nat. Neurosci.* 17 304–311. 10.1038/nn.3606 24362760 PMC4044925

[B124] Kinney J. W. Bemiller S. M. Murtishaw A. S. Leisgang A. M. Salazar A. M. Lamb B. T. (2018). Inflammation as a central mechanism in Alzheimer’s disease. *Alzheimers Dement.* 4 575–590. 10.1016/j.trci.2018.06.014 PMC6214864 30406177

[B125] Kishnani P. S. Spiridigliozzi G. A. Heller J. H. Sullivan J. A. Doraiswamy P. M. Krishnan K. R. (2001). Donepezil for Down’s syndrome. *Am. J. Psychiatry* 158:143. 10.1176/appi.ajp.158.1.143 11136652

[B126] Kiss J. Schlumpf M. Balazs R. (1989). Selective retardation of the development of the basal forebrain cholinergic and pontine catecholaminergic nuclei in the brain of trisomy 16 mouse, an animal model of Down’s syndrome. *Brain Res. Dev. Brain Res.* 50 251–264. 10.1016/0165-3806(89)90201-0 2575464

[B127] Klunk W. E. Price J. C. Mathis C. A. Tsopelas N. D. Lopresti B. J. Ziolko S. K. (2007). Amyloid deposition begins in the striatum of presenilin-1 mutation carriers from two unrelated pedigrees. *J. Neurosci.* 27 6174–6184. 10.1523/jneurosci.0730-07.2007 17553989 PMC3265970

[B128] Koelliker A. (1896). *Handbuch der Gewebelehre des Menschen. Nervensystem des Menschen und der Thiere*, 6th Edn, Vol. 2. Leipzig: W. Engelmann.

[B129] Korbel J. O. Tirosh-Wagner T. Urban A. E. Chen X. N. Kasowski M. Dai L. (2009). The genetic architecture of Down syndrome phenotypes revealed by high-resolution analysis of human segmental trisomies. *Proc. Natl. Acad. Sci. U.S.A.* 106 12031–12036. 10.1073/pnas.0813248106 19597142 PMC2709665

[B130] Kosik K. S. Joachim C. L. Selkoe D. J. (1986). Microtubule-associated protein tau (tau) is a major antigenic component of paired helical filaments in Alzheimer disease. *Proc. Natl. Acad. Sci. U.S.A.* 83 4044–4048. 10.1073/pnas.83.11.4044 2424016 PMC323662

[B131] Lao P. J. Handen B. L. Betthauser T. J. Mihaila I. Hartley S. L. Cohen A. D. (2017). Longitudinal changes in amyloid positron emission tomography and volumetric magnetic resonance imaging in the nondemented Down syndrome population. *Alzheimers Dement.* 9 1–9. 10.1016/j.dadm.2017.05.001 28603769 PMC5454131

[B132] Lao P. J. Handen B. L. Betthauser T. J. Mihaila I. Hartley S. L. Cohen A. D. (2018). Alzheimer-like pattern of hypometabolism emerges with elevated amyloid-β burden in Down syndrome. *J. Alzheimers Dis.* 61 631–644. 10.3233/jad-170720 29254096 PMC5994924

[B133] Lejeune J. Turpin R. Gautier M. (1959). Le mogolisme, premier exemple d’aberration autosomique humaine. *Ann. Genet.* 1 41–49.

[B134] Li L. B. Chang K. H. Wang P. R. Hirata R. K. Papayannopoulou T. Russell D. W. (2012). Trisomy correction in down syndrome induced pluripotent stem cells. *Cell Stem Cell* 11 615–619. 10.1016/j.stem.2012.08.004 23084023 PMC3705773

[B135] Li X. J. Zhang X. Johnson M. A. Wang Z. B. Lavaute T. Zhang S. C. (2009). Coordination of sonic hedgehog and Wnt signaling determines ventral and dorsal telencephalic neuron types from human embryonic stem cells. *Development* 136 4055–4063. 10.1242/dev.036624 19906872 PMC2778748

[B136] Li Z. Y. Yu T. Morishima M. Pao A. LaDuca J. Conroy J. (2007). Duplication of the entire 22.9 Mb human chromosome 21 syntenic region on mouse chromosome 16 causes cardiovascular and gastrointestinal abnormalities. *Hum. Mol. Genet.* 16 1359–1366. 10.1093/hmg/ddm086 17412756

[B137] Lin S.-C. Nicolelis M. A. (2008). Neuronal ensemble bursting in the basal forebrain encodes salience irrespective of valence. *Neuron* 59 138–149. 10.1016/j.neuron.2008.04.031 18614035 PMC2697387

[B138] Liu P. P. Xie Y. Meng X. Y. Kang J. S. (2019). History and progress of hypotheses and clinical trials for Alzheimer’s disease. *Signal Transduct. Target. Ther.* 4:29. 10.1038/s41392-019-0063-8 PMC6799833 31637009

[B139] Liu Y. Liu H. Sauvey C. Yao L. Zarnowska E. D. Zhang S. C. (2013a). Directed differentiation of forebrain GABA interneurons from human pluripotent stem cells. *Nat. Protocols* 8 1670–1679. 10.1038/nprot.2013.106 23928500 PMC4121169

[B140] Liu Y. Weick J. P. Liu H. Krencik R. Zhang X. Ma L. (2013b). Medial ganglionic eminence–like cells derived from human embryonic stem cells correct learning and memory deficits. *Nat. Biotechnol.* 31 440–447. 10.1038/nbt.2565 23604284 PMC3711863

[B141] Lockrow J. Boger H. Bimonte-Nelson H. Granholm A. C. (2011). Effects of long-term memantine on memory and neuropathology in Ts65Dn mice, a model for Down syndrome. *Behav. Brain Res.* 221 610–622. 10.1016/j.bbr.2010.03.036 20363261 PMC2928411

[B142] Lockrow J. Prakasam A. Huang P. Bimonte-Nelson H. Sambamurti K. Granholm A. C. (2009). Cholinergic degeneration and memory loss delayed by vitamin E in a Down syndrome mouse model. *Exp. Neurol.* 216 278–289. 10.1016/j.expneurol.2008.11.021 19135442 PMC2704550

[B143] Lott I. T. Head E. (2019). Dementia in Down syndrome: unique insights for Alzheimer disease research. *Nat. Rev. Neurol.* 15 135–147. 10.1038/s41582-018-0132-6 30733618 PMC8061428

[B144] Lott I. T. Head E. Doran E. Busciglio J. (2006). Beta-amyloid, oxidative stress and down syndrome. *Curr. Alzheimer Res.* 3 521–528. 10.2174/156720506779025305 17168651

[B145] Lu H. E. Yang Y. C. Chen S. M. Su H. L. Huang P. C. Tsai M. S. (2013). Modeling neurogenesis impairment in down syndrome with induced pluripotent stem cells from Trisomy 21 amniotic fluid cells. *Exp. Cell Res.* 319 498–505. 10.1016/j.yexcr.2012.09.017 23041301

[B146] MacLean G. A. Menne T. F. Guo G. J. Sanchez D. J. Park I. H. Daley G. Q. (2012). Altered hematopoiesis in trisomy 21 as revealed through in vitro differentiation of isogenic human pluripotent cells. *Proc. Natl. Acad. Sci. U.S.A.* 109 17567–17572. 10.1073/pnas.1215468109 23045682 PMC3491455

[B147] Mandas A. Mereu R. M. Catte O. Saba A. Serchisu L. Costaggiu D. (2014). Cognitive impairment and age-related vision disorders: their possible relationship and the evaluation of the use of aspirin and statins in a 65 years-and-over Sardinian population. *Front. Aging Neurosci.* 6:309. 10.3389/fnagi.2014.00309 25426067 PMC4224124

[B148] Mattson M. P. Magnus T. (2006). Ageing and neuronal vulnerability. *Nat. Rev. Neurosci.* 7 278–294. 10.1038/nrn1886 16552414 PMC3710114

[B149] McNaughton D. Knight W. Guerreiro R. Ryan N. Lowe J. Poulter M. (2012). Duplication of amyloid precursor protein (APP), but not prion protein (PRNP) gene is a significant cause of early onset dementia in a large UK series. *Neurobiol. Aging* 33 426.e13–e21. 10.1016/j.neurobiolaging.2010.10.010 PMC3657692 21193246

[B150] Meda L. Cassatella M. A. Szendrei G. I. Otvos L. Jr. Baron P. Villalba M. (1995). Activation of microglial cells by beta-amyloid protein and interferon-gamma. *Nature* 374 647–650. 7715705 10.1038/374647a0

[B151] Menendez M. (2005). Down syndrome, Alzheimer’s disease and seizures. *Brain Dev.* 27 246–252. 15862185 10.1016/j.braindev.2004.07.008

[B152] Mengel D. Liu W. Glynn R. J. Selkoe D. J. Strydom A. Lai F. (2020). Dynamics of plasma biomarkers in Down syndrome: the relative levels of A beta 42 decrease with age, whereas NT1 tau and NfL increase. *Alzheimers Res. Ther.* 12:27. 10.1186/s13195-020-00593-7 PMC7081580 32192521

[B153] Mesulam M. Shaw P. Mash D. Weintraub S. (2004). Cholinergic nucleus basalis tauopathy emerges early in the aging-MCI-AD continuum. *Ann. Neurol.* 55 815–828. 10.1002/ana.20100 15174015

[B154] Mesulam M. M. Mufson E. J. Levey A. I. Wainer B. H. (1983a). Cholinergic innervation of cortex by the basal forebrain: cytochemistry and cortical connections of the septal area, diagonal band nuclei, nucleus basalis (substantia innominata), and hypothalamus in the rhesus monkey. *J. Comp. Neurol.* 214 170–197. 10.1002/cne.902140206 6841683

[B155] Mesulam M.M. Mufson E. J. Wainer B. Levey A. (1983b). Central cholinergic pathways in the rat: an overview based on an alternative nomenclature (Ch1–Ch6). *Neuroscience* 10 1185–1201. 10.1016/0306-4522(83)90108-2 6320048

[B156] Meynert T. (1872). *Handbuch der Lehre von den Geweben des Menschen und der Thiere*, Vol. 1 ed. Stricker S. (Leipzig: W. Engelmann), 694–808.

[B157] Michaud M. Balardy L. Moulis G. Gaudin C. Peyrot C. Vellas B. (2013). Proinflammatory cytokines, aging, and age-related diseases. *J. Am. Med. Dir. Assoc.* 14 877–882. 23792036 10.1016/j.jamda.2013.05.009

[B158] Miettinen R. A. Kalesnykas G. Koivisto E. H. (2002). Estimation of the total number of cholinergic neurons containing estrogen receptor-alpha in the rat basal forebrain. *J. Histochem. Cytochem.* 50 891–902. 10.1177/002215540205000703 12070268

[B159] Mitsushima D. Sano A. Takahashi T. (2013). A cholinergic trigger drives learning-induced plasticity at hippocampal synapses. *Nat. Commun.* 4:2760. 10.1038/ncomms3760 PMC3831287 24217681

[B160] Mori H. (1997). The biological significance of neuropathological lesions in Alzheimer’s disease. *Neurobiol. Aging* 18 379–382. 10.1016/s0197-4580(97)00050-x 9330966

[B161] Mou X. N. Wu Y. B. Cao H. H. Meng Q. Z. Wang Q. H. Sun C. C. (2012). Generation of disease-specific induced pluripotent stem cells from patients with different karyotypes of Down syndrome. *Stem Cell Res. Ther.* 3:12. 10.1186/scrt105 PMC3392774 22512921

[B162] Mufson E. Counts S. Che S. Ginsberg S. (eds) (2004). cDNA array and quantitative PCR analysis of neurotrophin receptor transcripts in cholinergic basal forebrain neurons in people with mild cognitive impairment (MCI) and Alzheimer’s disease. *Proc. Soc. Neurosci.* 30, 335–336.

[B163] Mufson E. J. Bothwell M. Hersh L. B. Kordower J. H. (1989). Nerve growth factor receptor immunoreactive profiles in the normal, aged human basal forebrain: colocalization with cholinergic neurons. *J. Comp. Neurol.* 285 196–217. 10.1002/cne.902850204 2547849

[B164] Mufson E. J. Conner J. M. Kordower J. H. (1995). Nerve growth factor in Alzheimer’s disease: defective retrograde transport to nucleus basalis. *Neuroreport* 6 1063–1066. 10.1097/00001756-199505090-00028 7632896

[B165] Mufson E. J. Ginsberg S. D. Ikonomovic M. D. DeKosky S. T. (2003). Human cholinergic basal forebrain: chemoanatomy and neurologic dysfunction. *J. Chem. Neuroanat.* 26 233–242. 10.1016/s0891-0618(03)00068-1 14729126

[B166] Mufson E. J. Li J. M. Sobreviela T. Kordower J. H. (1996). Decreased trkA gene expression within basal forebrain neurons in Alzheimer’s disease. *Neuroreport* 8 25–29. 10.1097/00001756-199612200-00006 9051746

[B167] Muñoz S. S. Engel M. Balez R. Do-Ha D. Cabral-da-Silva M. C. Hernández D. (2020). A simple differentiation protocol for generation of induced pluripotent stem cell-derived basal forebrain-like cholinergic neurons for Alzheimer’s disease and frontotemporal dementia disease modeling. *Cells* 9:2018. 10.3390/cells9092018 32887382 PMC7564334

[B168] Murray A. Letourneau A. Canzonetta C. Stathaki E. Gimelli S. Sloan-Bena F. (2015). Brief report: isogenic induced pluripotent stem cell lines from an adult with mosaic Down syndrome model accelerated neuronal ageing and neurodegeneration. *Stem Cells* 33 2077–2084. 10.1002/stem.1968 25694335 PMC4737213

[B169] Nardone R. Marth R. Ausserer H. Bratti A. Tezzon F. (2006). Reduced short latency afferent inhibition in patients with Down syndrome and Alzheimer-type dementia. *Clin. Neurophysiol.* 117 2204–2210. 10.1016/j.clinph.2006.07.134 16931146

[B170] Naumann T. Casademunt E. Hollerbach E. Hofmann J. Dechant G. Frotscher M. (2002). Complete deletion of the neurotrophin receptor p75NTRLeads to long-lasting increases in the number of basal forebrain cholinergic neurons. *J. Neurosci.* 22 2409–2418. 10.1523/jneurosci.22-07-02409.2002 11923404 PMC6758339

[B171] Nukina N. Ihara Y. (1986). One of the antigenic determinants of paired helical filaments is related to tau Protein. *J. Biochem.* 99 1541–1544. 10.1093/oxfordjournals.jbchem.a135625 2423512

[B172] Ovchinnikov D. A. Korn O. Virshup I. Wells C. A. Wolvetang E. J. (2018). The Impact of APP on Alzheimer-like pathogenesis and gene expression in Down syndrome iPSC-derived neurons. *Stem Cell Rep.* 11 32–42. 10.1016/j.stemcr.2018.05.004 29861166 PMC6066957

[B173] Papavassiliou P. York T. P. Gursoy N. Hill G. Nicely L. V. Sundaram U. (2009). The phenotype of persons having mosaicism for trisomy 21/Down syndrome reflects the percentage of trisomic cells present in different tissues. *Am. J. Med. Genet. Part A* 149a 573–583. 10.1002/ajmg.a.32729 19291777 PMC3707311

[B174] Parikh V. Sarter M. (2008). Cholinergic mediation of attention: contributions of phasic and tonic increases in prefrontal cholinergic activity. *Ann. N. Y. Acad. Sci.* 1129 225–235. 10.1196/annals.1417.021 18591483

[B175] Park I. H. Arora N. Huo H. Maherali N. Ahfeldt T. Shimamura A. (2008a). Disease-specific induced pluripotent stem cells. *Cell* 134 877–886. 18691744 10.1016/j.cell.2008.07.041 PMC2633781

[B176] Park I. H. Zhao R. West J. A. Yabuuchi A. Huo H. Ince T. A. (2008b). Reprogramming of human somatic cells to pluripotency with defined factors. *Nature* 451 141–146. 10.1038/nature06534 18157115

[B177] Perez S. E. Miguel J. C. He B. Malek-Ahmadi M. Abrahamson E. E. Ikonomovic M. D. (2019). Frontal cortex and striatal cellular and molecular pathobiology in individuals with Down syndrome with and without dementia. *Acta Neuropathol.* 137 413–436. 10.1007/s00401-019-01965-6 30734106 PMC6541490

[B178] Perluigi M. Butterfield D. A. (2012). Oxidative stress and Down syndrome: a route toward Alzheimer-Like dementia. *Curr. Gerontol. Geriatr. Res.* 2012:724904. 10.1155/2012/724904 PMC3235450 22203843

[B179] Perry E. K. Johnson M. Kerwin J. M. Piggott M. A. Court J. A. Shaw P. J. (1992). Convergent cholinergic activities in aging and Alzheimers-disease. *Neurobiol. Aging* 13 393–400. 10.1016/0197-4580(92)90113-c 1625768

[B180] Pipino C. Mukherjee S. David A. L. Blundell M. P. Shaw S. W. Sung P. (2014). Trisomy 21 mid-trimester amniotic fluid induced pluripotent stem cells maintain genetic signatures during reprogramming: implications for disease modeling and cryobanking. *Cell. Reprogram.* 16 331–344. 10.1089/cell.2013.0091 25162836

[B181] Pitchers K. K. Phillips K. B. Jones J. L. Robinson T. E. Sarter M. (2017). Diverse roads to relapse: a discriminative cue signaling cocaine availability is more effective in renewing cocaine seeking in goal trackers than sign trackers and depends on basal forebrain cholinergic activity. *J. Neurosci.* 37 7198–7208. 10.1523/jneurosci.0990-17.2017 28659281 PMC5546399

[B182] Powers B. E. Kelley C. M. Velazquez R. Ash J. A. Strawderman M. S. Alldred M. J. (2017). Maternal choline supplementation in a mouse model of Down syndrome: effects on attention and nucleus basalis/substantia innominata neuron morphology in adult offspring. *Neuroscience* 340 501–514. 10.1016/j.neuroscience.2016.11.001 27840230 PMC5177989

[B183] Powers B. E. Velazquez R. Kelley C. M. Ash J. A. Strawderman M. S. Alldred M. J. (2016). Attentional function and basal forebrain cholinergic neuron morphology during aging in the Ts65Dn mouse model of Down syndrome. *Brain Struct. Funct.* 221 4337–4352. 10.1007/s00429-015-1164-y 26719290 PMC4929047

[B184] Prasher V. P. Farrer M. J. Kessling A. M. Fisher E. M. C. West R. J. Barber P. C. (1998). Molecular mapping of Alzheimer-type dementia in Down’s syndrome. *Ann. Neurol.* 43 380–383.9506555

[B185] Presson A. P. Partyka G. Jensen K. M. Devine O. J. Rasmussen S. A. McCabe L. L. (2013). Current estimate of down syndrome population prevalence in the United States. *J. Pediatr.* 163 1163–1168. 10.1016/j.jpeds.2013.06.013 23885965 PMC4445685

[B186] Price D. Kitt C. A. Hedreen J. Whitehouse P. Struble R. Cork L. (1986). “Basal forebrain cholinergic systems in primate brain: anatomical organization and role in the pathology of aging and dementia,” in *Dynamics of Cholinergic Function: Advances in Behavioral Biology*, ed. Hanin I. (Boston, MA: Springer), 235–242. 10.1007/978-1-4684-5194-8_21

[B187] Rafii M. S. Ances B. M. Schupf N. Krinsky-McHale S. J. Mapstone M. Silverman W. (2020). The AT (N) framework for Alzheimer’s disease in adults with Down syndrome. *Alzheimers Dement.* 12:e12062. 10.1002/dad2.12062 PMC7588820 33134477

[B188] Rafii M. S. Lukic A. S. Andrews R. D. Brewer J. Rissman R. A. Strother S. C. (2017). PET imaging of tau pathology and relationship to amyloid, longitudinal MRI, and cognitive change in Down syndrome: results from the Down syndrome biomarker initiative (DSBI). *J. Alzheimers Dis.* 60 439–450. 10.3233/jad-170390 28946567

[B189] Real R. Peter M. Trabalza A. Khan S. Smith M. A. Dopp J. (2018). In vivo modeling of human neuron dynamics and Down syndrome. *Science* 362:eaau1810. 10.1126/science.aau1810 30309905 PMC6570619

[B190] Reeves R. H. Irving N. G. Moran T. H. Wohn A. Kitt C. Sisodia S. S. (1995). A mouse model for Down syndrome exhibits learning and behaviour deficits. *Nat. Genet.* 11 177–184. 10.1038/ng1095-177 7550346

[B191] Remes A. M. Laru L. Tuominen H. Aalto S. Kemppainen N. Mononen H. (2008). Carbon 11–labeled Pittsburgh Compound B positron emission tomographic amyloid imaging in patients with APP locus duplication. *Arch. Neurol.* 65 540–544. 10.1001/archneur.65.4.540 18413480

[B192] Rong S. Li Y. Li B. Nie K. Zhang P. Cai T. (2021). Meynert nucleus-related cortical thinning in Parkinson’s disease with mild cognitive impairment. *Quant. Imaging Med. Surg.* 11 1554–1566. 10.21037/qims-20-444 33816191 PMC7930701

[B193] Rovelet-Lecrux A. Frebourg T. Tuominen H. Majamaa K. Campion D. Remes A. M. (2007). APP locus duplication in a Finnish family with dementia and intracerebral haemorrhage. *J. Neurol. Neurosurg. Psychiatry.* 78 1158–1159. 10.1136/jnnp.2006.113514 17442758 PMC2117532

[B194] Rovelet-Lecrux A. Hannequin D. Raux G. Le Meur N. Laquerrière A. Vital A. (2006). APP locus duplication causes autosomal dominant early-onset Alzheimer disease with cerebral amyloid angiopathy. *Nat. Genet.* 38 24–26. 10.1038/ng1718 16369530

[B195] Rusted J. (1994). Cholinergic blockade and human information processing: are we asking the right questions? *J. Psychopharmacol. (Oxf. Engl.)* 8 54–59. 10.1177/026988119400800109 22298481

[B196] Sago H. Carlson E. J. Smith D. J. Kilbridge J. Rubin E. M. Mobley W. C. (1998). Ts1Cje, a partial trisomy 16 mouse model for Down syndrome, exhibits learning and behavioral abnormalities. *Proc. Natl. Acad. Sci. U.S.A.* 95 6256–6261. 10.1073/pnas.95.11.6256 9600952 PMC27649

[B197] Sakurai T. Nagata R. Yamanaka A. Kawamura H. Tsujino N. Muraki Y. (2005). Input of orexin/hypocretin by a genetically encoded neurons revealed tracer in mice. *Neuron* 46 297–308. 10.1016/j.neuron.2005.03.010 15848807

[B198] Salehi A. Delcroix J. D. Belichenko P. V. Zhan K. Wu C. Valletta J. S. (2006). Increased App expression in a mouse model of Down’s syndrome disrupts NGF transport and causes cholinergic neuron degeneration. *Neuron* 51 29–42. 10.1016/j.neuron.2006.05.022 16815330

[B199] Salehi A. Delcroix J. D. Swaab D. F. (2004). Alzheimer’s disease and NGF signaling. *J. Neural Trans.* 111 323–345. 10.1007/s00702-003-0091-x 14991458

[B200] Sanders N. C. Williams D. K. Wenger G. R. (2009). Does the learning deficit observed under an incremental repeated acquisition schedule of reinforcement in Ts65Dn mice, a model for Down syndrome, change as they age? *Behav. Brain Res.* 203 137–142. 10.1016/j.bbr.2009.04.031 19409933 PMC2700176

[B201] Sarter M. Bruno J. P. Givens B. (2003). Attentional functions of cortical cholinergic inputs: what does it mean for learning and memory? *Neurobiol. Learn. Mem.* 80 245–256. 10.1016/s1074-7427(03)00070-4 14521867

[B202] Sawada M. Sawada H. Nagatsu T. (2008). Effects of aging on neuroprotective and neurotoxic properties of microglia in neurodegenerative diseases. *Neurodegener. Dis.* 5 254–256. 10.1159/000113717 18322405

[B203] Saxena S. Caroni P. (2011). Selective neuronal vulnerability in neurodegenerative diseases: from stressor thresholds to degeneration. *Neuron* 71 35–48. 10.1016/j.neuron.2011.06.031 21745636

[B204] Schmitz T. W. Duncan J. (2018). Normalization and the cholinergic microcircuit: a unified basis for attention. *Trends Cogn. Sci.* 22 422–437. 10.1016/j.tics.2018.02.011 29576464

[B205] Schmitz T. W. Soreq H. Poirier J. Spreng R. N. (2020). Longitudinal basal forebrain degeneration interacts with TREM2/C3 biomarkers of inflammation in presymptomatic Alzheimer’s disease. *J. Neurosci.* 40 1931–1942. 10.1523/jneurosci.1184-19.2019 31915256 PMC7046458

[B206] Scott B. S. Becker L. E. Petit T. L. (1983). Neurobiology of Down’s syndrome. *Prog. Neurobiol.* 21 199–237. 6197719 10.1016/0301-0082(83)90002-3

[B207] Selkoe D. J. (1991). The molecular pathology of alzheimers-disease. *Neuron* 6 487–498. 1673054 10.1016/0896-6273(91)90052-2

[B208] Selkoe D. J. (2002). Alzheimer’s disease is a synaptic failure. *Science* 298 789–791. 12399581 10.1126/science.1074069

[B209] Selkoe D. J. Hardy J. (2016). The amyloid hypothesis of Alzheimer’s disease at 25years. *EMBO Mol. Med.* 8 595–608. 27025652 10.15252/emmm.201606210 PMC4888851

[B210] Sepulcre J. Grothe M. J. d’Oleire Uquillas F. Ortiz-Terán L. Diez I. Yang H.-S. (2018). Neurogenetic contributions to amyloid beta and tau spreading in the human cortex. *Nat. Med.* 24 1910–1918. 10.1038/s41591-018-0206-4 30374196 PMC6518398

[B211] Sheedy F. J. Grebe A. Rayner K. J. Kalantari P. Ramkhelawon B. Carpenter S. B. (2013). CD36 coordinates NLRP3 inflammasome activation by facilitating intracellular nucleation of soluble ligands into particulate ligands in sterile inflammation. *Nat. Immunol.* 14 812–820. 10.1038/ni.2639 23812099 PMC3720827

[B212] Shi Y. Kirwan P. Smith J. Robinson H. P. Livesey F. J. (2012). Human cerebral cortex development from pluripotent stem cells to functional excitatory synapses. *Nat. Neurosci.* 15 477–486, S1. 22306606 10.1038/nn.3041 PMC3882590

[B213] Shin M. Besser L. M. Kucik J. E. Lu C. Siffel C. Correa A. (2009). Prevalence of Down syndrome among children and adolescents in 10 regions of the United States. *Pediatrics* 124 1565–1571. 10.1542/peds.2009-0745 19948627

[B214] Sinai A. Mokrysz C. Bernal J. Bohnen I. Bonell S. Courtenay K. (2018). Predictors of age of diagnosis and survival of Alzheimer’s disease in Down syndrome. *J. Alzheimers Dis.* 61 717–728. 29226868 10.3233/JAD-170624 PMC6004911

[B215] Sleegers K. Brouwers N. Gijselinck I. Theuns J. Goossens D. Wauters J. (2006). APP duplication is sufficient to cause early onset Alzheimer’s dementia with cerebral amyloid angiopathy. *Brain* 129 2977–2983. 10.1093/brain/awl203 16921174

[B216] Snyder H. M. Bain L. J. Brickman A. M. Carrillo M. C. Esbensen A. J. Espinosa J. M. (2020). Further understanding the connection between Alzheimer’s disease and Down syndrome. *Alzheimers Dement.* 16 1065–1077. 32544310 10.1002/alz.12112 PMC8865308

[B217] Sobol M. Klar J. Laan L. Shahsavani M. Schuster J. Anneren G. (2019). Transcriptome and proteome profiling of neural induced pluripotent stem cells from individuals with Down syndrome disclose dynamic dysregulations of key pathways and cellular functions. *Mol. Neurobiol.* 56 7113–7127. 10.1007/s12035-019-1585-3 30989628 PMC6728280

[B218] Solari N. Hangya B. (2018). Cholinergic modulation of spatial learning, memory and navigation. *Eur. J. Neurosci.* 48 2199–2230. 10.1111/ejn.14089 30055067 PMC6174978

[B219] Startin C. M. Ashton N. J. Hamburg S. Hithersay R. Wiseman F. K. Mok K. Y. (2019). Plasma biomarkers for amyloid, tau, and cytokines in Down syndrome and sporadic Alzheimer’s disease. *Alzheimers Res. Ther.* 11:26. 10.1186/s13195-019-0477-0 PMC6429702 30902060

[B220] Strydom A. Coppus A. Blesa R. Danek A. Fortea J. Hardy J. (2018). Alzheimer’s disease in Down syndrome: an overlooked population for prevention trials. *Alzheimers Dement.* 4 703–713. 10.1016/j.trci.2018.10.006 30581976 PMC6296162

[B221] Sussel L. Marin O. Kimura S. Rubenstein J. L. (1999). Loss of Nkx2.1 homeobox gene function results in a ventral to dorsal molecular respecification within the basal telencephalon: evidence for a transformation of the pallidum into the striatum. *Development* 126 3359–3370. 10.1242/dev.126.15.3359 10393115

[B222] Swaminathan S. Huentelman M. J. Corneveaux J. J. Myers A. J. Faber K. M. Foroud T. (2012). Analysis of copy number variation in Alzheimer’s disease in a cohort of clinically characterized and neuropathologically verified individuals. *PLoS One* 7:e50640. 10.1371/journal.pone.0050640 23227193 PMC3515604

[B223] Sweeney J. E. Hohmann C. F. Oster-Granite M. L. Coyle J. T. (1989). Neurogenesis of the basal forebrain in euploid and trisomy 16 mice: an animal model for developmental disorders in Down syndrome. *Neuroscience* 31 413–425. 10.1016/0306-4522(89)90384-9 2529451

[B224] Takahashi K. Tanabe K. Ohnuki M. Narita M. Ichisaka T. Tomoda K. (2007). Induction of pluripotent stem cells from adult human fibroblasts by defined factors. *Cell* 131 861–872. 10.1016/j.cell.2007.11.019 18035408

[B225] Tao Y. Zhang S. C. (2016). Neural subtype specification from human pluripotent stem cells. *Cell Stem Cell* 19 573–586. 10.1016/j.stem.2016.10.015 27814479 PMC5127287

[B226] Tayebati S. K. Cecchi A. Martinelli I. Carboni E. Amenta F. (2019). Pharmacotherapy of Down’s syndrome: when and which? *CNS Neurol. Disord. Drug Targets* 18 750–757. 10.2174/1871527318666191114092924 31724517

[B227] Terry R. D. Masliah E. Salmon D. P. Butters N. DeTeresa R. Hill R. (1991). Physical basis of cognitive alterations in Alzheimer’s disease: synapse loss is the major correlate of cognitive impairment. *Ann. Neurol.* 30 572–580. 10.1002/ana.410300410 1789684

[B228] Thal D. R. Rüb U. Orantes M. Braak H. (2002). Phases of Aβ-deposition in the human brain and its relevance for the development of AD. *Neurology* 58 1791–1800. 10.1212/wnl.58.12.1791 12084879

[B229] Thomson J. A. Itskovitz-Eldor J. Shapiro S. S. Waknitz M. A. Swiergiel J. J. Marshall V. S. (1998). Embryonic stem cell lines derived from human blastocysts. *Science* 282 1145–1147. 10.1126/science.282.5391.1145 9804556

[B230] Thonberg H. Fallström M. Björkström J. Schoumans J. Nennesmo I. Graff C. (2011). Mutation screening of patients with Alzheimer disease identifies APP locus duplication in a Swedish patient. *BMC Res. Notes* 4:476. 10.1186/1756-0500-4-476 22044463 PMC3216298

[B231] Tramutola A. Lanzillotta C. Barone E. Arena A. Zuliani I. Mosca L. (2018). Intranasal rapamycin ameliorates Alzheimer-like cognitive decline in a mouse model of Down syndrome. *Transl. Neurodegener.* 7:28. 10.1186/s40035-018-0133-9 PMC6218962 30410750

[B232] Tudorascu D. Laymon C. Zammit M. Minhas D. Anderson S. Ellison P. (2020). Relationship of amyloid beta and neurofibrillary tau deposition in Neurodegeneration in Aging Down Syndrome (NiAD) study at baseline. *Alzheimers Dement.* 6:e12096. 10.1002/trc2.12096 PMC7602678 33163613

[B233] Tudorascu D. L. Anderson S. J. Minhas D. S. Yu Z. Comer D. Lao P. (2019). Comparison of longitudinal Aβ in nondemented elderly and Down syndrome. *Neurobiol. Aging* 73 171–176. 10.1016/j.neurobiolaging.2018.09.030 30359879 PMC6251757

[B234] van Reekum R. Black S. E. Conn D. Clarke D. (1997). Cognition-enhancing drugs in dementia: a guide to the near future. *Can. J. Psychiatry* 42(Suppl. 1) 35s–50s. 9220128

[B235] Villemagne V. L. Ataka S. Mizuno T. Brooks W. S. Wada Y. Kondo M. (2009). High striatal amyloid β-peptide deposition across different autosomal Alzheimer disease mutation types. *Arch. Neurol.* 66 1537–1544. 20008660 10.1001/archneurol.2009.285

[B236] Visser F. E. Aldenkamp A. P. van Huffelen A. C. Kuilman M. Overweg J. van W. J. (1997). Prospective study of the prevalence of Alzheimer-type dementia in institutionalized individuals with Down syndrome. *Am. J. Ment. Retard.* 101 400–412. 9017086

[B237] Wang H. F. Liu F. C. (2001). Developmental restriction of the LIM homeodomain transcription factor Islet-1 expression to cholinergic neurons in the rat striatum. *Neuroscience* 103 999–1016. 10.1016/s0306-4522(00)00590-x 11301207

[B238] Weick J. P. Held D. L. Bonadurer G. F. Doers M. E. Liu Y. Maguire C. (2013). Deficits in human trisomy 21 iPSCs and neurons. *Proc. Natl. Acad. Sci. U.S.A.* 110 9962–9967. 10.1073/pnas.1216575110 23716668 PMC3683748

[B239] Weinberger N. M. (2007). Associative representational plasticity in the auditory cortex: a synthesis of two disciplines. *Learn. Memory.* 14 1–16. 10.1101/lm.421807 17202426 PMC3601844

[B240] White K. G. Ruske A. C. (2002). Memory deficits in Alzheimer’s disease: the encoding hypothesis and cholinergic function. *Psychonom. Bull. Rev.* 9 426–437. 10.3758/bf03196301 12412885

[B241] Whitehouse P. J. Price D. L. Clark A. W. Coyle J. T. DeLong M. R. (1981). Alzheimer disease: evidence for selective loss of cholinergic neurons in the nucleus basalis. *Ann. Neurol.* 10 122–126. 10.1002/ana.410100203 7283399

[B242] Whitehouse P. J. Price D. L. Struble R. G. Clark A. W. Coyle J. T. Delon M. R. (1982). Alzheimer’s disease and senile dementia: loss of neurons in the basal forebrain. *Science* 215 1237–1239. 10.1126/science.7058341 7058341

[B243] Wilcock D. M. (2012). Neuroinflammation in the aging Down syndrome brain; lessons from Alzheimer’s disease. *Curr. Gerontol. Geriatr. Res.* 2012:170276. 10.1155/2012/170276 PMC3290800 22454637

[B244] Wiseman F. K. Al-Janabi T. Hardy J. Karmiloff-Smith A. Nizetic D. Tybulewicz V. L. J. (2015). A genetic cause of Alzheimer disease: mechanistic insights from Down syndrome. *Nat. Rev. Neurosci.* 16 564–574. 10.1038/nrn3983 26243569 PMC4678594

[B245] Wiseman F. K. Pulford L. J. Barkus C. Liao F. Portelius E. Webb R. (2018). Trisomy of human chromosome 21 enhances amyloid-beta deposition independently of an extra copy of APP. *Brain* 141 2457–2474. 10.1093/brain/awy159 29945247 PMC6061702

[B246] Woolf N. J. (1991). Cholinergic systems in mammalian brain and spinal cord. *Prog. Neurobiol.* 37 475–524. 10.1016/0301-0082(91)90006-m 1763188

[B247] Wu H. Williams J. Nathans J. (2014). Complete morphologies of basal forebrain cholinergic neurons in the mouse. *Elife* 3:e02444. 10.7554/eLife.02444 PMC4038840 24894464

[B248] Xu Q. Guo L. Moore H. Waclaw R. R. Campbell K. Anderson S. A. (2010). Sonic hedgehog signaling confers ventral telencephalic progenitors with distinct cortical interneuron fates. *Neuron* 65 328–340. 10.1016/j.neuron.2010.01.004 20159447 PMC2868511

[B249] Xu Q. Tam M. Anderson S. A. (2008). Fate mapping Nkx2.1-lineage cells in the mouse telencephalon. *J. Comp. Neurol.* 506 16–29. 10.1002/cne.21529 17990269

[B250] Xu Q. Wonders C. P. Anderson S. A. (2005). Sonic hedgehog maintains the identity of cortical interneuron progenitors in the ventral telencephalon. *Development* 132 4987–4998. 10.1242/dev.02090 16221724

[B251] Xu W. Fang F. Ding J. Wu C. (2018). Dysregulation of Rab5-mediated endocytic pathways in Alzheimer’s disease. *Traffic* 19 253–262. 10.1111/tra.12547 29314494 PMC5869093

[B252] Xu W. Weissmiller A. M. White J. A. Fang F. Wang X. Y. Wu Y. W. (2016). Amyloid precursor protein-mediated endocytic pathway disruption induces axonal dysfunction and neurodegeneration. *J. Clin. Investig.* 126 1815–1833. 10.1172/jci82409 27064279 PMC4855914

[B253] Yan R. Vassar R. (2014). Targeting the β secretase BACE1 for Alzheimer’s disease therapy. *Lancet Neurol.* 13 319–329. 10.1016/s1474-4422(13)70276-x 24556009 PMC4086426

[B254] Yankner B. A. Duffy L. K. Kirschner D. A. (1990). Neurotrophic and neurotoxic effects of amyloid beta-protein – reversal by tachykinin neuropeptides. *Science* 250 279–282. 10.1126/science.2218531 2218531

[B255] Yates C. M. Simpson J. Maloney A. F. J. Gordon A. Reid A. H. (1980). Alzheimer-like cholinergic deficiency in down syndrome. *Lancet* 2:979. 10.1016/s0140-6736(80)92137-6 6107618

[B256] Yiannopoulou K. G. Papageorgiou S. G. (2020). Current and future treatments in Alzheimer disease: an update. *J. Central Nerv. Syst. Dis.* 12:1179573520907397. 10.1177/1179573520907397 PMC7050025 32165850

[B257] Yu J. Vodyanik M. A. Smuga-Otto K. Antosiewicz-Bourget J. Frane J. L. Tian S. (2007). Induced pluripotent stem cell lines derived from human somatic cells. *Science* 318 1917–1920. 18029452 10.1126/science.1151526

[B258] Yu T. Li Z. Y. Jia Z. P. Clapcote S. J. Liu C. H. Li S. M. (2010). A mouse model of Down syndrome trisomic for all human chromosome 21 syntenic regions. *Hum. Mol. Genet.* 19 2780–2791. 10.1093/hmg/ddq179 20442137 PMC2893810

[B259] Yue W. Li Y. Y. Zhang T. Jiang M. Qian Y. Zhang M. (2015). ESC-Derived basal forebrain cholinergic neurons ameliorate the cognitive symptoms associated with Alzheimer’s disease in mouse models. *Stem Cell Rep.* 5 776–790. 10.1016/j.stemcr.2015.09.010 26489896 PMC4649256

[B260] Zaborszky L. Csordas A. Mosca K. Kim J. Gielow M. R. Vadasz C. (2015). Neurons in the Basal Forebrain Project to the cortex in a complex topographic organization that reflects corticocortical connectivity patterns: an experimental study based on retrograde tracing and 3D reconstruction. *Cereb. Cortex* 25 118–137. 10.1093/cercor/bht210 23964066 PMC4259277

[B261] Zaborszky L. Rosin D. L. Kiss J. (2004). Alpha-adrenergic receptor (alpha(2 A)) is colocalized in basal forebrain cholinergic neurons: a light and electron microscopic double immunolabeling study. *J. Neurocytol.* 33 265–276. 10.1023/b:neur.0000044188.67442.9d 15475682

[B262] Zammit M. D. Laymon C. M. Betthauser T. J. Cody K. A. Tudorascu D. L. Minhas D. S. (2020a). Amyloid accumulation in Down syndrome measured with amyloid load. *Alzheimers Dement.* 12:e12020. 10.1002/dad2.12020 PMC7233422 32435686

[B263] Zammit M. D. Laymon C. M. Tudorascu D. L. Hartley S. L. Piro-Gambetti B. Johnson S. C. (2020b). Patterns of glucose hypometabolism in Down syndrome resemble sporadic Alzheimer’s disease except for the putamen. *Alzheimers Dement.* 12:e12138. 10.1002/dad2.12138 PMC7804861 33490360

[B264] Zammit M. D. Tudorascu D. L. Laymon C. M. Hartley S. L. Zaman S. H. Ances B. M. (2021). PET measurement of longitudinal amyloid load identifies the earliest stages of amyloid-beta accumulation during Alzheimer’s disease progression in Down syndrome. *Neuroimage* 228 117728. 10.1016/j.neuroimage.2021.117728 33421595 PMC7953340

[B265] Zhang K. Fishel Ben Kenan R. Osakada Y. Xu W. Sinit R. S. Chen L. (2013). Defective axonal transport of Rab7 GTPase results in dysregulated trophic signaling. *J. Neurosci.* 33 7451–7462. 10.1523/jneurosci.4322-12.2013 23616551 PMC3722856

[B266] Zhao X. Bhattacharyya A. (2018). Human models are needed for studying human neurodevelopmental disorders. *Am. J. Hum. Genet.* 103 829–857. 10.1016/j.ajhg.2018.10.009 30526865 PMC6288051

[B267] Zhou Y. Sun Y. Ma Q.-H. Liu Y. (2018). Alzheimer’s disease: amyloid-based pathogenesis and potential therapies. *Cell Stress* 2:150. 10.15698/cst2018.07.143 31225482 PMC6551797

[B268] Zigman W. B. Schupf N. Sersen E. Silverman W. (1996). Prevalence of dementia in adults with and without Down syndrome. *Am. J. Ment. Retard.* 100 403–412. 8718994

