# Corrigendum: Basal Forebrain Cholinergic Neurons: Linking Down Syndrome and Alzheimer's Disease

**DOI:** 10.3389/fnagi.2021.742233

**Published:** 2021-08-05

**Authors:** Jose L. Martinez, Matthew D. Zammit, Nicole R. West, Bradley T. Christian, Anita Bhattacharyya

**Affiliations:** ^1^Cellular and Molecular Biology Graduate Program, University of Wisconsin, Madison, WI, United States; ^2^Waisman Center, University of Wisconsin, Madison, WI, United States; ^3^Department of Medical Physics, School of Medicine and Public Health, University of Wisconsin, Madison, WI, United States; ^4^Department of Psychiatry, School of Medicine and Public Health, University of Wisconsin, Madison, WI, United States; ^5^Department of Cellular and Regenerative Biology, School of Medicine and Public Health, University of Wisconsin, Madison, WI, United States

**Keywords:** basal forebrain cholinergic neurons, down syndrome, Alzheimer's disease, pluripotent stem cell, neurodegeneration

In the original article, we neglected to include the funder **Jérôme Lejeune Foundation**, to **Anita Bhattacharyya**.

The authors apologize for this error and state that this does not change the scientific conclusions of the article in any way. The original article has been updated.

## Publisher's Note

All claims expressed in this article are solely those of the authors and do not necessarily represent those of their affiliated organizations, or those of the publisher, the editors and the reviewers. Any product that may be evaluated in this article, or claim that may be made by its manufacturer, is not guaranteed or endorsed by the publisher.

